# Six-hour time-restricted feeding inhibits lung cancer progression and reshapes circadian metabolism

**DOI:** 10.1186/s12916-023-03131-y

**Published:** 2023-11-03

**Authors:** Dan Shi, Gaofeng Fang, Qianyao Chen, Jianling Li, Xiongzhong Ruan, Xuemei Lian

**Affiliations:** 1 Center for Lipid Research, Key Laboratory of Molecular Biology for Infectious Diseases (Ministry of Education), The Second Affiliated Hospital, Chongqing Medical University, Chongqing, 400016 P.R. China; 2 Department of Nutrition and Food Hygiene, School of Public Health, Chongqing Medical University, Chongqing, 400016 P.R. China; 3 Research Center for Environment and Population Health, School of Public Health, Chongqing Medical University, Chongqing, 400016 P. R. China; 4 Nutrition Innovation Platform-Sichuan and Chongqing, School of Public Health, Chongqing Medical University, Chongqing, China

**Keywords:** Time-restricted feeding, Lung cancer, Progression, Circadian rhythm, TIM, Autophagy

## Abstract

**Background:**

Accumulating evidence has suggested an oncogenic effect of diurnal disruption on cancer progression. To test whether targeting circadian rhythm by dietary strategy suppressed lung cancer progression, we adopted 6-h time-restricted feeding (TRF) paradigm to elucidate whether and how TRF impacts lung cancer progression.

**Methods:**

This study used multiple lung cancer cell lines, two xenograft mouse models, and a chemical-treated mouse lung cancer model. Stable TIM-knockdown and TIM-overexpressing A549 cells were constructed. Cancer behaviors in vitro were determined by colony formation, EdU proliferation, wound healing, transwell migration, flow cytometer, and CCK8 assays. Immunofluorescence, pathology examinations, and targeted metabolomics were also used in tumor cells and tissues. mCherry-GFP-LC3 plasmid was used to detect autophagic flux.

**Results:**

We found for the first time that compared to normal ad libitum feeding, 6-h TRF inhibited lung cancer progression and reprogrammed the rhythms of metabolites or genes involved in glycolysis and the circadian rhythm in tumors. After TRF intervention, only timeless (TIM) gene among five lung cancer-associated clock genes was found to consistently align rhythm of tumor cells to that of tumor tissues. Further, we demonstrated that the anti-tumor effect upon TRF was partially mediated by the rhythmic downregulation of the TIM and the subsequent activation of autophagy. Combining TRF with TIM inhibition further enhanced the anti-tumor effect, comparable to treatment efficacy of chemotherapy in xenograft model.

**Conclusions:**

Six-hour TRF inhibits lung cancer progression and reshapes circadian metabolism, which is partially mediated by the rhythmic downregulation of the TIM and the subsequent upregulation of autophagy.

**Supplementary Information:**

The online version contains supplementary material available at 10.1186/s12916-023-03131-y.

## Introduction

Lung cancer is one of the most common malignant diseases globally, ranking second in cancer morbidity and first in cancer mortality worldwide [1]. Although tremendous advances in surgical techniques and neoadjuvant chemoradiotherapy have been made in recent years, the therapeutic prognosis of patients with lung cancer has only modestly improved, with a 5-year survival rate of 10–20% [2]. Recently, accumulating evidence has suggested a connection between circadian metabolism and cancer, specifically an oncogenic effect of diurnal disruption on cancer progression [3–5]. Circadian rhythm disruption by physiologic perturbation or genetic mutation of the circadian clock significantly shortens survival and drive growth and progression of lung cancer [6]. Circadian disruption enhances tumorigenesis in lung cancer model [7]. These observations indicate that targeting circadian metabolism may be an alternative and effective strategy for lung cancer prevention and treatment, although further investigation is required.

One way to target and reprogram circadian rhythm is to modulate the timing of food intake via a time-restricted feeding (TRF) diet; this dietary approach helps organisms align the timing of internal circadian metabolism to the external environment [8–10]. TRF, a form of intermittent fasting in which all nutrient intake is restricted to within typically ≤ 12 h of the day, accompanied by a longer daily fasting period without an overt attempt to impose energy restriction, has drawn considerable attention in the field of nutrition [8, 9]. Mounting evidence indicates that energy restriction has broad health benefits and remains the most robust dietary strategy to date for achieving antitumor effects in various contexts, but the results mainly pertain to model animals [11, 12]. Notably, energy restriction in model animals is frequently accompanied by a change in feeding behavior, with food being consumed within a few hours [13, 14]. Therefore, energy restriction and TRF appear to be mutually intertwined in enhancing the antitumor effect of energy restriction in model animals. Although data are limited to a few animal studies, recent studies have revealed that 4–12 h TRF might suppress the growth and progression of some tumors, including pancreatic adenocarcinoma, renal cancer, and breast cancer, and inhibit the metastasis of lung cancer [15–20]. However, whether TRF inhibits lung cancer growth and progression is still largely unknown, and the complex mechanisms by which TRF affects cancer risk and development remain to be elucidated.

Altered rhythmic metabolism and circadian gene expression appear to be key factors in anti-tumor effect of TRF [16, 18, 21]. TRF slows down osteosarcoma progression and reprogrammed rhythmic components in activity [18], as well as reverses the jet lag-driven alterations in clock genes expression [21]. Reinforcement of the circadian timekeeping system with TRF promotes 24-h rhythmic expressions of key genes, leading to growth inhibition of pancreatic adenocarcinoma [16]. Among circadian genes, the circadian regulator TIMELESS (TIM) is involved in tumor-related DNA replication [22, 23]. A few studies have linked TIM with lung cancer and reported a pro-proliferative role in lung cancer cells and a positive correlation with poor patient survival [24, 25]. However, whether TIM and its circadian expression are involved in lung cancer growth and progression in response to TRF have yet not been reported.

In this study, we aimed to elucidate whether and how 6-h TRF impacts lung cancer growth and progression and rewires circadian metabolism. We conducted a series of in vitro experiments using multiple lung cancer cell lines and in vivo experiments using two xenograft and a urethane-induced lung tumorigenesis mouse models under normal dietary conditions. We first demonstrated that 6-h TRF medium consistently inhibited the proliferation and migration of multiple lung cancer cell lines in vitro. Moreover, in both mouse models, 6-h TRF (access to normal chow for 6 h, 10 pm–4 am) delayed tumor initiation and growth in the absence of calorie restriction. Notably, TRF suppressed glycolysis, activated autophagy, and reprogrammed the rhythms of metabolites/genes involved in glycolysis and the circadian rhythm in tumors. We further demonstrated that TIM regulated autophagy as part of the mechanism by which TRF mediated tumor suppression. Finally, TRF combined with TIM inhibition further enhanced the anti-tumor efficacy in the xenograft model, indicating the potential of the combination regimen for therapeutic translation to the clinic.

## Materials and methods

### Cells and reagents

The human lung cancer cell lines A549 were obtained from the American Type Culture Collection (ATCC, Shanghai, China); NCI-H460 were obtained from the Chinese Academy of Sciences Cell Bank (Shanghai, China); NCI-H1975, PC9, and NCI-H1299 were obtained from the Chinese Tissue Culture Collections (Zhejiang, China). Cell lines were cultured in RPMI 1640 medium (C11875500BT, Gibco, NY, USA) supplemented with 10% fetal bovine serum (FBS) (04–001-1ACS, BI, Haemek, Israel) and 100 U/ml penicillin–streptomycin mix (C0222, Beyotime, Shanghai, China) at 37 °C in an atmosphere with 5% CO_2_ and 95% air. Cell identity has been authenticated using STR profiling and no mycoplasma contamination was detected in cells.

### Lentivirus construction and stable cell line selection

The following constructs were used: TIM overexpression plasmid of YOE-LV005-TIMELESS (Ubigene, Guangzhou, China), YOE-LV005-Ctrl empty vector (Ubigene, Guangzhou, China), TIM shRNA-1 constructs of the LV-U6 > hTIMELESS [shRNA #1]-PGK > EGFP/T2A/Puro (Cyagen Biosciences, Jiangsu, China), TIM shRNA-2 constructs of the LV-U6 > hTIMELESS [shRNA #2]-PGK > EGFP/T2A/Puro (Cyagen Biosciences, Jiangsu, China), LV-U6 > Scramble-shRNA-PGK > EGFP/T2A/Puro empty vector (Cyagen Biosciences, Jiangsu, China), GPI overexpression plasmid of PDS279_pL-CMV-GFP-ccdB-puro-GPI (Tsingke, Beijing, China), PDS279_pL-CMV-GFP-ccdB-puro-blank empty vector (Tsingke, Beijing, China), GPI shRNA constructs of the VP013-U6-MCS-CMV-ZsGreen-PGK-PURO-GPI-shrna (Tsingke, Beijing, China), VP013-U6-MCS-CMV-ZsGreen-PGK-PURO-blank empty vector (Tsingke, Beijing, China). Sequences of sh-TIM1 and sh-TIM2 are 5’-GCATCCTCCATCTTGCCAAATCTCGAGATTTGGCAAGATGGAGGATGC-3’ and 5’-CAGGCCATTGTTTCTGGTAATCTCGAGATTACCAGAAACAATGGCC-TG-3’. Sequence of GPI shRNA is 5’-UUGUGAUGCAGACCCUUCCTT-3’. Lentiviral particles were produced by co-transfecting the gag/pol or rev plasmids and TIM/ GPI overexpression/ shRNA plasmids or empty vectors into HEK293T cells using the Lipofectamine 2000 Reagent (11668–027, Invitrogen, CA, USA) according to the instructions. Stable cell lines were established by transfection with the lentiviral particles followed by puromycin (HY-B1743A, MCE, Shanghai, China) or hygromycin B (HY-B0490, MCE, Shanghai, China) selection.

### TRF and reagent treatment in vitro

Cells in the control group were incubated with normal control medium consisting of glucose-free RPMI 1640 medium (11879020, Gibco, NY, USA) supplemented with 10% FBS (04–001-1ACS, BI, Haemek, Israel), 2 g/L glucose (ST491-100 ml, Beyotime, China) in a day, whereas cells in the TRF group were treated with TRF paradigm consisting of normal control medium for 6 h and glucose-free RPMI 1640 medium (11879020, Gibco, NY, USA) supplemented with 0% FBS (04–001-1ACS, BI, Haemek, Israel) for 18 h in a day. Cells in the control and TRF groups were washed twice with PBS before switching to other medium or their fresh culture medium, and the washing time in the control group was the same as that of the TRF group.

For the TRF combined with rapamycin (53123–88-9, MCE, Shanghai, China) group, cells were treated with 15 nM rapamycin for 6 h during the normal medium phase, continuing 15 nM rapamycin treatment for 6 h during glucose-free, FBS-free medium phase in a day. For the TRF combined with CQ (54–05-7, MCE, Shanghai, China) group, cells were treated with 30 µM CQ for 6 h merely during the normal medium phase in a day.

### Animals and diets

All animals were maintained under pathogen-free conditions with a 12:12 h light/dark cycle in an environmentally constant room at a 21 ± 2 °C temperature and 50% ± 5% humidity with free access to water. All protocols were approved by the Medical Ethics Committee of Chongqing Medical University (ethics number: IACUC-CQMU-2023–0006, Chongqing, China) and were performed in accordance with the Guide for the Care and Use of Laboratory Animals.

In vivo subcutaneous xenograft model: Five-week-old male BALB/c nude mice were purchased from Beijing HFK Bioscience Co., Ltd. (Beijing, China). A total of 6 × 10^6^ or 1 × 10^7^ cells (A549, A549 TIM OE-Ctrl empty vector, A549 OE-TIM, A549 TIM sh-Ctrl empty vector, A549 sh-TIM, A549 GPI sh-Ctrl empty vector, or A549 sh-GPI) or 2 × 10^6^ H460 cells were subcutaneously implanted in the right flank. Once the tumors became palpable, the mice were randomly assigned to a control group or TRF group with or without carboplatin treatment. The mice in the control group had ad libitum access to normal chow throughout the day, whereas the animals in the TRF group were allowed access to food between ZT14 (2 h after lights off) and ZT20 (4 h before lights on) daily. The TRF mice were manually provided and removed with food pellet in each cage at ZT14 and ZT20 time points every day, respectively. All used cages (control group and TRF cages) were replaced with new sterilized cages every 1 week to stay cleanliness. Carboplatin was administered by intraperitoneal injection (50 mg/kg, HY-17393, MCE, Shanghai, China) once per week at the initiation of TRF diet. Food intake and body weight were recorded weekly in the morning by weighing. Tumor volume was measured with digital calipers every 3 days for 27 to 33 consecutive days and calculated using the formula *V* = (length × width^2^)/2. Mice were euthanized with sodium pentobarbital (90 mg/kg, i.p.) at the end of the experiment or when the tumor volume exceeded 1500 mm^3^.

Urethane-induced lung tumorigenesis model: Five-week-old C57BL/6 J male mice were purchased from Vital River Laboratory Animal Technology Company Ltd. (Beijing, China) and housed under pathogen-free conditions in individually ventilated caging (IVC) systems. As described by our previous study with slight modification [26], after 3 days of adaptation, the mice were randomly grouped and fed an ad libitum control diet or TRF (food access between ZT14 and ZT20) for 3 weeks. Thereafter, the mice in the control and TRF groups were randomized for treatment with saline or urethane (1 mg/g, 76,607, Sigma‒Aldrich Inc., USA) by intraperitoneal injection every day for 10 consecutive weeks. After a 15-week latency period, the mice were euthanized with sodium pentobarbital (90 mg/kg, i.p.), and lung tumorigenesis was assessed. During the entire study period, food intake and body weight were recorded weekly in the morning.

### Cells collection for gene expression detection in vitro

Cells were subjected to control and TRF paradigms for 2 consecutive days before a 2-h 50% horse serum shock. Then, the cells were switched to the normal control medium for sample collection at 4-h intervals from ZT1 (11 PM) to ZT21 (7 PM). In the in vitro study, ZT0 was set as the time point at which the TRF intervention ended. Cells in the control and TRF groups were washed twice with PBS before switching to other medium or their fresh culture medium, and the washing time in the control group was the same as that of the TRF group. Three to four replicates per time point were analyzed for gene expression detection.

### Colony formation assay

Lung cancer cell lines, including the A549, H460, H1975, PC9, and H1299 cell lines and stable overexpression or knockdown cell lines were plated in six-well plates at a density of 400–800 cells per well. The cells were treated with normal control or TRF paradigm with or without treatment with rapamycin or CQ for 2–3 days and then cultured with normal medium for 8–11 days. Next, the cells were washed with PBS, fixed in 4% paraformaldehyde for 20 min at room temperature, dyed with crystal violet for 20 min, and photographed with a cell phone. Relative colony area was measured using Fiji software. Three independent replicates were performed.

### EdU proliferation assay

A total of 4 × 10^6^ cells were seeded on coverslips and subjected to control or TRF paradigm with or without treatment with rapamycin or CQ for 2 consecutive days. Then, BeyoClick™ EdU-488 Kits (C0071S, Beyotime, Shanghai, China) or BeyoClick™ EdU-555 Kits (C0075S, Beyotime, Shanghai, China) were used to detect cell proliferation according to the manufacturer’s protocols. Briefly, 10 μM EdU reagent was added to cells and incubation for 2 h at 37 °C, followed by fixation in 4% paraformaldehyde for 15 min and by permeabilization with 0.3% Triton- × 100 for 15 min at room temperature. Then, cells were washed with PBS and incubated with Click Additive Solution for 30 min at room temperature, protected from light. Further, cells were stained nuclear using Hoechst. Staining was observed under a confocal laser scanning microscope (Leica TCS SP2, Leica, Weztlar, Germany) after sealing with antifade mounting medium (P0126, Beyotime, Shanghai, China). Cell proliferation ability was quantified using Leica LAS X software. Randomly chosen 4 field of views were evaluated for each sample. Three independent experiments were carried out.

### Wound healing assay

A total of 2 × 10^5^–3 × 10^5^ cells were inoculated into six-well plates and grown to over 80% confluence overnight. Then, a scratch was made through the confluent cell monolayer using a 200-μl pipette tip. The cells were treated with control or TRF paradigm for 2 days. During this period, photographs of the same scratched areas were taken using an optical microscope (EVOS XL Core, Thermo Finnigan, Austin, USA) at 0, 24, and 48 h. Migration distances were quantified with Fiji. Three independent replicates were carried out.

### Transwell migration assay

Cell culture inserts (#0286, Corning, NY, USA) containing a transparent PET membrane with an 8-μm pore size were used to measure cell migration. After cells were treated with control or TRF paradigms for 2 days, 2 × 10^4^ to 4 × 10^4^ cells suspended in 200 µL serum-free RPMI 1640 medium were seeded in the upper chambers, and 600 μL RPMI 1640 medium containing 15% FBS was added to the lower chambers. After 24 h of incubation, the cells on the lower surface of the membrane were fixed in 4% paraformaldehyde for 20 min at room temperature, followed by staining with 0.1% crystal violet for 20 min. At least 3–5 fields were photographed for each chamber using an optical microscope (EVOS XL Core, Thermo Finnigan, Austin, USA).

### Immunofluorescence

A549 cells were seeded on coverslips, subjected to control or TRF paradigm for 2 days, and then switched to normal control medium. Then, the coverslips were collected every 8 h over a 24-h period, fixed in 4% paraformaldehyde, and permeabilized with 0.5% Triton X-100. Then, the coverslips were blocked in QuickBlock™ Blocking Buffer for Immunol Staining (P0260, Beyotime, Shanghai, China) for 20 min, followed by sequential incubations with a mouse anti-GPI monoclonal antibody (TA501128, OriGene, Wuxi, China, dilution 1:500, overnight at 4 °C) and Alexa Fluor 647-labeled goat anti-mouse IgG (H + L) (A0473, Beyotime, Shanghai, China, dilution 1:500, 1 h at 37 °C). The coverslips were counterstained with Hoechst for 5–10 min to label the nuclei and visualized by confocal laser scanning microscopy (Leica TCS SP2, Leica, Weztlar, Germany).

### Cell cycle and apoptosis analyses

For cell cycle analysis, adherent cells seeded in six-well plates were treated with control or TRF paradigm for 2 days, and then, the cells were collected, washed with precooled PBS, and fixed with precooled 75% ethanol. Cell cycle analysis was conducted by propidium iodide (PI) staining according to the manufacturer’s protocols (P4170, Sigma Aldrich, St Louis, USA). Cell cycle assessments were performed with a CytoFLEX flow cytometer (Beckman Coulter, CA, USA) and CytExpert V2.3.0.84 software (Beckman Coulter, USA). Three replicates for each sample were detected.

For apoptosis analysis, adherent cells treated with control feeding or TRF for 2 days were collected, washed, and resuspended in precooled PBS. FITC Annexin V Apoptosis Detection Kit I (E-CK-A211, E-CK-A252, Elabscience, Wuhan, China) was used according to the manufacturer’s instructions. Annexin V staining was used to identify early apoptosis, while PI and DAPI staining were used to identify late apoptosis. Cell apoptosis assessments were performed with a CytoFLEX flow cytometer (Beckman Coulter, CA, USA) and CytExpert V2.3.0.84 software (Beckman Coulter, USA). Three replicates for each sample were detected.

### CCK-8 assay

In total, 10^4^ cells per well were inoculated in 96-well plates and treated with TRF, rapamycin, CQ, TRF + RA, or TRF + CQ for 2 days. Cell viability was detected by the CCK-8 method (HY-K0301, MCE, Shanghai, China) according to the manufacturer’s instructions. Briefly, 10 μg CCK-8 solution was added to each well and incubated at 37 °C for 1 h. The OD values were assessed at 450 nm by an Agilent BioTek Synergy H1 multimode reader.

### Collection of blood and tissues

At the experimental endpoint, mice were fasted starting at ZT20 (4 h before lights on) and then randomly anesthetized with chloral hydrate at 4-h or 6-h intervals from ZT1 (11 PM) to ZT21 (7 PM). Blood samples were collected immediately by cardiac puncture following anesthesia and collected in plain tubes. Serum was collected by centrifugation (3000 r/min for 15 min at 4 °C) and stored at − 80 °C for further analysis. Animal tissues (tumor, lung, liver and muscles) were collected and weighted by an electronic scale; one tissue specimen was fixed for pathological examination, and the remaining specimen was stored at − 80 °C for further analysis. The muscles included the hind legs gastrocnemius and soleus muscles of mice.

### Pathology examinations and quantification

Paraformaldehyde-fixed paraffin sections of tumor and lung tissues were stained with hematoxylin and eosin (H&E). Histological examinations were conducted by pathologists blinded to the groups. In addition, the tumor tissues were further scored for mitotic cell. For immunohistochemistry assay, tumor slides were de-paraffinized with xylene for 30 min and hydrated in gradient concentration of alcohols of 100–70% for 5 min followed by washing with water. Thereafter, the slides were soaked in heated antigen retrieval solution (10 mM Sodium citrate, pH 8.0 or 10 mM EDTA) for 20 min and then were allowed to be cool to room temperature. UltraSensitiveTM SP (Mouse/Rabbit) IHC Kit (KIT-9710, MXB, Fuzhou, China) was then applied to block endogenous peroxidase and nonspecific staining and amplify the signal of primarily antibodies, according to the manufacturers’ protocol. The following primarily antibodies were used for staining: mouse monoclonal anti-PCNA antibody (MAB-0145, MXB, 100 µl, 60 min at room temperature); mouse monoclonal anti-CD31 antibody (MAB-0031, MXB, 100 µl, 60 min at room temperature); rabbit monoclonal anti-Ki67 antibody (ET1609-34, HUABIO, hangzhou,1:200 dilution, overnight at 4 °C); rabbit monoclonal anti-Timeless antibody (ab109512, Abcam, USA, 1:600 dilution, overnight at 4 °C); rabbit monoclonal anti-LC3B antibody (#3868, CST, USA, 1:200 dilution, overnight at 4 °C); and rabbit monoclonal anti-P62 antibody (WL02385, Wanglei, China, 1:2000 dilution, overnight at 4 °C). The DAB kit (KIT-0014, MXB, Fuzhou, China) was applied as a chromogenic agent and Meyer’s hematoxylin for counterstaining. Sections were photographed using a pannoramic Scanner (3DHISTECH, Budapest, Hungary). At least 4–8 fields were scored for each slide. Images were quantified by Fiji (https://imagej.net/Fiji) and positive staining was extracted using trainable Weka segmentation plugin.

### Lung tumor enumeration

After animals were euthanized, 4% paraformaldehyde was injected into the lungs and the entire lung tissues were harvested. Thereafter, lung tumors were counted under a dissecting microscope by three experimental researchers blinded to the groups.

### Blood chemistry measurement

Serum parameters of GLU (A154-2–1), TCHO (A111-1–1), TG (A110-1–1), LDL-C (A113-1–1), HDL-C(A112-1–1) (NJJCBIO, Naijing, China), insulin (SP14098, Spbio, Wuhan, China), and IGF1 (SP13776, Spbio, Wuhan, China) were assayed with commercial kits according to the manufacturers’ instructions (*n* = 4–6 per group at each time).

### Indirect calorimetry

As described by our previous study [27], the indirect calorimetry (TSE, German) test was performed on male mice at a temperature (21 ± 2 °C) and humidity (50% ± 5%) with a 12:12 h light/dark cycle in the environmentally constant room (*n* = 8 per group). Mice were placed individually into the respirometry cages designed with food hoppers and water bottles on the top, and the cage bottom was covered with bedding material. Mice were allowed free access to food and water and were granted to adapt to the respirometry cages for 2 h prior to the start of experiment. Stable air pressure and flow rates were monitored daily to guarantee the acquirement of meaningful calorimetry results. The experiment started at 9 am and was consecutively monitored for 3 days. Measurements, such as the values of oxygen consumption (VO2), carbon dioxide production (VCO2), respiratory exchange ratio (RER) and energy expenditure (EE), were output to the computer at 3-min intervals. We analyzed data from the last two days to attempt to minimize bias caused by the new environment. Above results were analyzed with adjustment for body weight.

### Western blot analysis

Total proteins from tumor tissues or cells were extracted with RIPA lysis buffer (P0013B, P0013C, Beyotime, Shanghai, China) and protease inhibitor (P1006, Beyotime, Shanghai, China) plus phenylmethanesulfonyl fluoride (PMSF) (ST506, Beyotime, Shanghai, China). Antibodies were used for western blotting as follows: rabbit monoclonal anti-Timeless antibody (ab109512, Abcam, Boston, USA); mouse monoclonal anti-GPI (TA501128, ORIGENE, MD, USA); rabbit monoclonal anti-LC3B antibody (#3868, CST, Beverly, MA, USA); and rabbit monoclonal anti-P62 antibody (WL02385, Wanglei, Shanghai, China; 18420–1-AP, Proteintech, Chicago, USA) and β-actin (TA-09, ZSGB-BIO, Beijing, China); goat anti-rabbit secondary antibody (ZB-2301, ZSGB-BIO, Beijing, China), and goat anti-mouse secondary antibody (ZB2305, ZSGB-BIO, Beijing, China). BeyoECL Moon kit (P0018FS, Beyotime, Shanghai, China) was used to detect the signal.

### Quantitative real-time PCR

Total RNA was isolated from tissues or cells with TRIzol reagent (9109, Takara, Kyoto, Japan) according to the manufacturer’s protocols. RNA was reverse transcribed to cDNA using the High-Capacity cDNA Reverse Transcription Kit (RR047A-5, Takara, Kyoto, Japan). Real-time PCR was carried out using the SYBR Green PCR Master Mix (HY-K0511, MCE, Shanghai, China) and the 7500 FAST Real-time PCR System (BIO-RAD, California, USA). All gene primers were obtained from Sangon Biotech (Sangon Biotech, Jiangsu, China). All primer sequences were proved to be specific and effective prior to use. Data were normalized to the gene expression of human β-actin or mouse β-actin. Details of primer sequences used are presented in Table S8.

### Measurement of energy-related metabolites

Targeted metabolomics analysis in the tumor tissues was conducted using liquid chromatography-tandem mass spectrometry (LC–MS/ MS) with the aid of Applied Protein Technology company and was described as follows:

#### Chemicals and reagents

Acetonitrile (I592230123, Merck, Darmstadt, Germany), methanol (144282, Merck, Darmstadt, Germany), formic acid (06450, Sigma-Aldrich, St Louis, USA), CH3COONH4 (70221, Sigma-Aldrich, St Louis, USA), and metabolic standards (Sigma-Aldrich, St Louis, USA).

#### Sample preparation

One hundred milligrams of tumor tissues were homogenized with 1 mL liquid mixture containing H2O/methanol/acetonitrile (1:2:2, v/v/v) by homogenizer and sonication and centrifuged at 13,000* g* for 15 min at 4 °C. The supernatant was collected and dried in a vacuum centrifuge followed by re-dissolved in 100 μL water/acetonitrile (1:1, v/v). The solution was mixed and transferred to an autosampler vial for LC–MS/ MS analysis. Quality control (QC) samples were prepared by mixing all samples equally.

#### Chromatography

Chromatographic separation was carried out using an Agilent 1290 Infinity LC UHPLC (Agilent Technologies, CA, USA) with ACQUITY UPLC BEH Amide column (2.1 × 100 mm, 1.7 μm, Waters, Milford, USA). A 4 µL solution was injected into the 45 °C column at a flow rate of 400 uL/ min through an autosampler. The mobile phase included solutions A (15 mM CH3COONH4 in water) and solution B (pure acetonitrile). The elution gradients were as follows: 90–40% B for 0–18 min, 40–90% B for 18–18.1 min, and 90%B for 18.1–23 min. The QC samples were injected every 5 samples.

#### Mass spectrometry

Mass spectrometry was performed using a 5500 QTRAP mass spectrometer (AB Sciex, Framingham, USA) equipped with electrospray ionization (ESI) in the negative mode. The conditions were set as follows: the source temperature was 450 °C; ion Source Gas1 was 45; ion Source Gas2 was 45; curtain gas was 30, and ionSapary Voltage Floating was 4500 V. MRM mode was adopted to detect the ion pair.

#### Data processing

The Multiquant software was applied to extract retention time (RT) and peak area. The retention time was corrected by the standards of metabolites, and the metabolites were identified.

### Autophagic flux analysis

mCherry-GFP-LC3 plasmid, which expressed LC3 fused with mCherry and GFP, was used to detect autophagic flux. mCherry-GFP-LC3 plasmid was obtained from the laboratory of professor Lin Yong. Cells were seeded on coverslips and transfected with plasmid using the Lipo8000™ Reagent (C0533, Beyotime, Shanghai, China) for 6 h, followed by control or TRF intervention for 2 days. Coverslips were fixed and permeabilizated by paraformaldehyde and Triton X-100 (P0096, Beyotime, Shanghai, China), respectively. Nuclear was stained by Hoechst (C0071S, Beyotime, Shanghai, China). Staining results were observed under a confocal laser scanning microscope (Leica TCS SP2, Leica, Weztlar, Germany) after sealing with antifade mounting medium (P0126, Beyotime, Shanghai, China). GFP (green fluorescent signal), but not mCherry (red fluorescent signal), is highly susceptible to the acidic environment of lysosomes, which leads to the loss of fluorescence. Thus, colocalization of both GFP and mCherry proteins (yellow) denotes an autophagosome, while only localization of mCherry fluorescence but not GFP (red) denotes an autolysosome. Three independent experiments were carried out.

### Transmission electron microscopy

Cells treated with control or TRF for 2 consecutive days were collected and followed by fixed using 4% glutaraldehyde overnight at 4 °C. The experiment was performed with the aid of staffs from electron microscope laboratory of Chongqing Medical University. Briefly, sections were dehydrated in gradient alcohol and embedded using EPON 812. Sections were made and stained with 2% uranyl acetate and 0.3% lead citrate. Images were taken using Hitachi-7500 transmission electron microscope (Hitachi, Tokyo, Japan).

### Statistical analysis

Unless otherwise stated, values were expressed as mean ± SEM or mean ± SD, as appropriate. Statistical calculations were performed with SPSS 22.0 software (IBM SPSS, Chicago, IL), GraphPad Software (version 7), or R (version 3.5.2). Significance was determined by using two-way ANOVA, one-way ANOVA with various post hoc tests, unpaired student *t*-tests, nonparametric tests, or Pearson correlation test, as appropriate. Genes or metabolites expressions over 24 h were analyzed and visualized using the circacompare package or metacycle in R, as appropriate. The value of period was set 24 h in this study. MESOR, amplitude, and phase for the dual-oscillating genes or metabolites were further compared when there was rhythmicity in both groups. Phase-shift was calculated by method described by previous study [28]. Briefly, if genes or metabolites in both control and TRF groups were circadian (both *P* value < 0.05), then the absolute value of phase difference upon the two groups was calculated as |phase in Control—phase in TRF|; if |phase in Control—phase in TRF|< 12, then phase shift = 24—|phase in Control—phase in TRF|, or phase shift =|phase in Control—phase in TRF|. A *P* value < 0.05 was interpreted as statistically significant.

## Results

### TRF inhibits lung cancer cell proliferation and migration in vitro

**Fig. 1:**
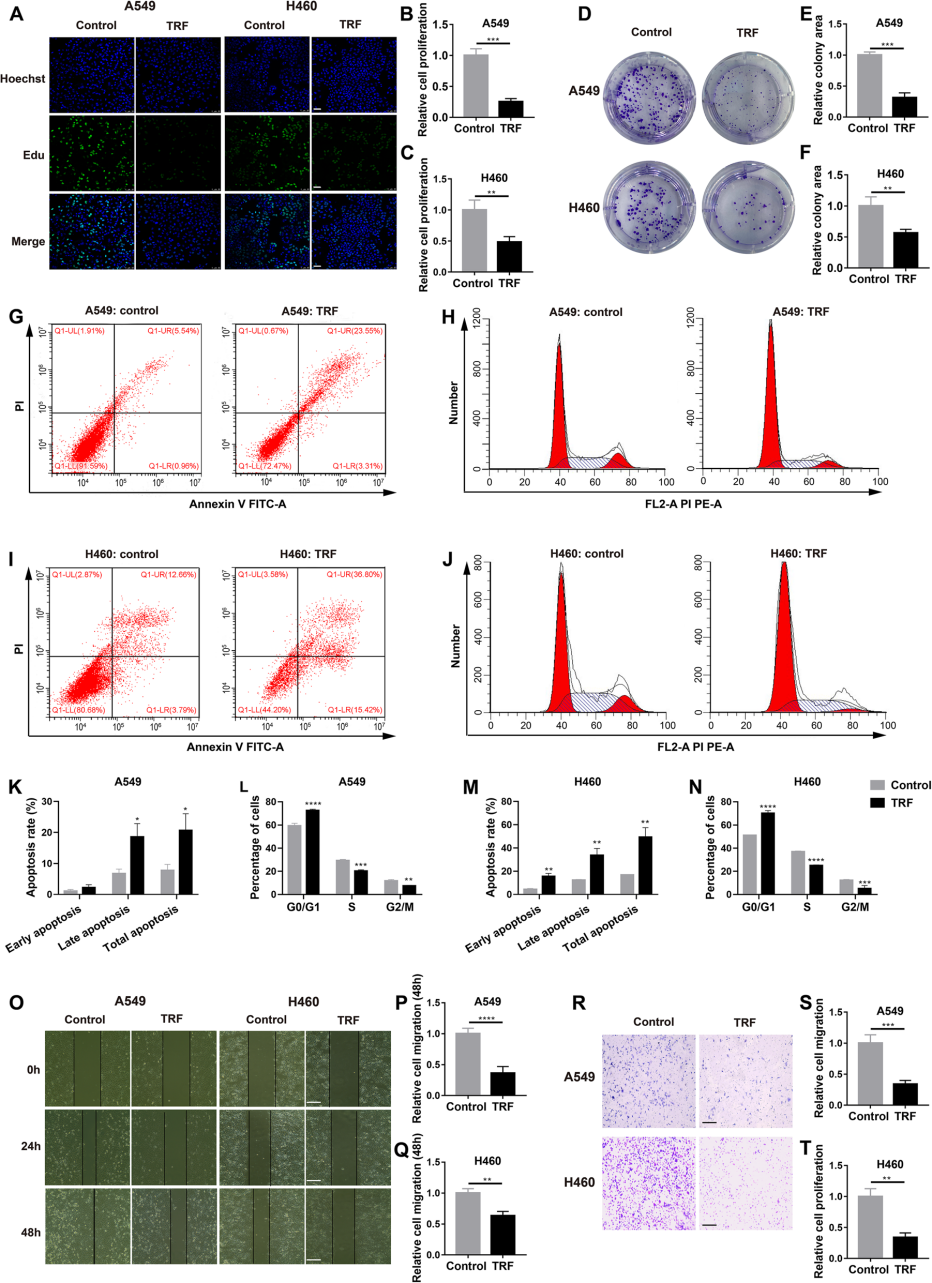
TRF inhibits lung cancer cell proliferation and migration in vitro. **A** Representative images of EdU assay to detect the proliferation of A549 and H460 cells in the control and time-restricted feeding (TRF) groups (scale bar = 50 µm). Control intervention: normal control medium in a day, TRF intervention: normal control medium for 6 h and glucose-free and FBS-free medium for 18 h in a day. Repeat the intervention for 2 days. **B**,** C** Quantification of the EdU results for **B** A549 and **c** H460 cells (*n* = 3). **D**–**F** Colony formation assay to determine the proliferation upon TRF with **D** representative images and quantification by **E** A549 and **F** H460 cells (*n* = 3). **G**–**N** Flow cytometry assay. Annexin V staining for early apoptosis and PI staining for late apoptosis and cell cycle distribution. **G**–**J** Representative images showing the effect of TRF on **G** apoptosis and **H** cell cycle distribution in the A549 cell. **I**–**J** Representative images showing the **I** apoptosis and **J** cell cycle distribution of H460 cells treated with TRF. **K**,** L** Quantification of **K** apoptosis and **L** the cell cycle in A549 cells (*n* = 3). **M**,** N** Quantification of **M** apoptosis and **N** the cell cycle in H460 cells (*n* = 3). **O**–**Q** Wound healing assay with **O** representative images (scale bar = 100 µm) and quantitative analysis performed with **P** A549 or **Q** H460 cells (*n* = 4). **R**–**T** Transwell migration assays with **R** representative images (scale bar = 100 µm), and quantitative analysis performed with **S** A549 or **T** H460 cells (*n* = 3–4). Data were analyzed by a two-tailed Student’s *t* test or two-way ANOVA with Tukey’s post hoc test. Expression relative to the control. Error bars, when present, show the SD. *P < 0.05; **P < 0.01; ***P < 0.001; ****P < 0.0001

To determine the effects of TRF on cancer behaviors, we first aimed to determine the changes in cancer cell line activities in proliferation and migration assays with 6-h TRF-mimicking paradigm for two consecutive days. Cells in the control group were incubated with normal control medium consisting of glucose-free RPMI 1640 medium supplemented with 10% FBS and 2 g/L glucose in a day, whereas cells in the TRF group were treated with 6-h TRF-mimicking paradigm consisting of normal control medium for 6 h and glucose-free and serum-free RPMI 1640 medium for 18 h during the 24-h period. As indicated by an EdU assay, 6-h TRF-mimicking paradigm significantly inhibited the proliferation of A549 cells (Fig. 1A, B) and H460 cells (Fig. 1A, C) compared to control treatment, representing the most common lung cancer cell lines. The TRF groups had relatively fewer colony-forming cells than the control groups, as indicated in colony formation assays for both the A549 cell line (Fig. 1D, E) and the H460 cell line (Fig. 1D, F), suggesting that TRF plays a role in suppressing cell proliferation.

To investigate the mechanism by which TRF inhibits cell proliferation, we assessed the effects of 6-h TRF-mimicking paradigm on apoptosis and the cell cycle with a flow cytometry assay with the two cell lines. 6-h TRF-mimicking paradigm increased the number of late and total apoptotic cells and induced the proportion of cells arrested in G0/G1 compared to control treatment (Fig. 1G-N).

Furthermore, to explore the role of TRF in cell migration, wound healing and Transwell migration assays were performed. Six-hour TRF-mimicking paradigm significantly reduced lung cancer cell migration relative to control treatment in both the A549 (Fig. 1O, P, R, S) and H460 cell lines (Fig. 1O, Q, R, T). Similar results were also obtained for other different subtypes of lung cancer cell lines (H1975, PC9, H1299) subjected to 6-h TRF-mimicking paradigm (Additional file 1: Fig. S1). Thus, taken together, these results demonstrate that TRF inhibits lung cancer cell proliferation and migration in vitro.

### TRF inhibits lung cancer growth in xenograft lung tumorigenesis mouse models

**Fig. 2:**
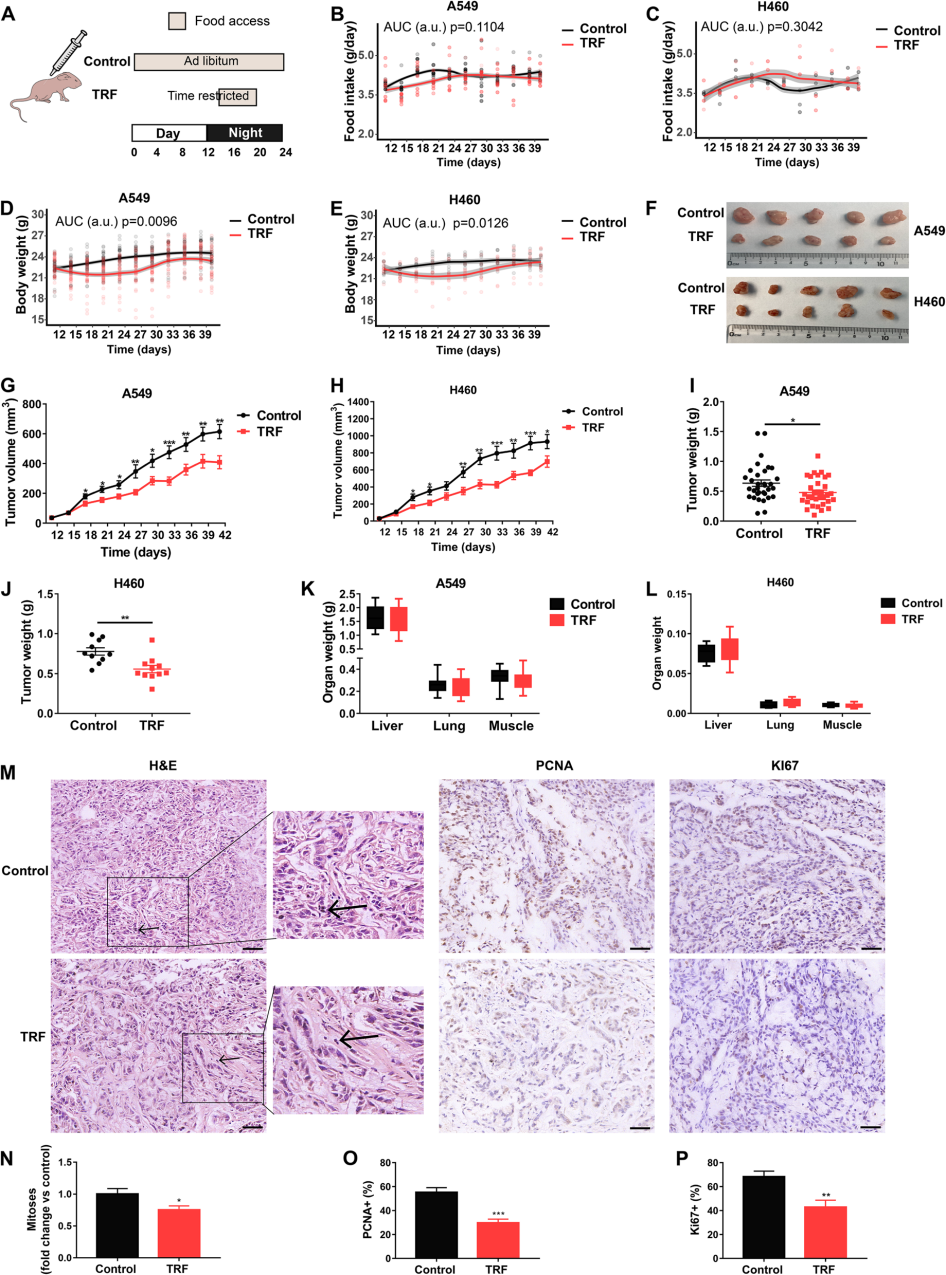
TRF attenuates lung cancer progression in two mouse xenograft models. **A** Schematic outline of the experimental design. **B, C** Food intake trajectories from the **B** A549 xenograft model and **C** H460 xenograft model (*n* = 30 mice per group). The dots on the graphs represent the values of individual mice. The gray outline represents the confidence interval. **D, E** Body weight trajectories from the **D** A549 xenograft model (*n* = 37–39 mice per group) and **E** H460 xenograft model (*n* = 10–11 mice per group). **F** Photograph of dissected tumors derived from the two xenograft models. **G, H** Tumor volume from the **G** A549 xenograft model (*n* = 37–39 mice per group) and **H** H460 xenograft model (*n* = 10–11 mice per group). **I–J** Tumor weight from the **I** A549 xenograft model (*n* = 33 mice per group) and **J** H460 xenograft model (*n* = 10–11 mice per group) at the end of the study. **K, L** Organ weight from the **K** A549 xenograft model (*n* = 33 mice per group) and **L** H460 xenograft model (*n* = 10–11 mice per group). Organ mass values are expressed as the organ weight relative to total body weight. **M** Histopathology. Representative H&E, PCNA, and Ki67 staining of tumor tissue from A549 xenograft model (scale bar = 50 µm), and the images of H&E-stained tumors on the right is an enlargement of the images on the left. The arrows indicate the mitoses. **N–P** Quantification of **N** mitoses, **O** PCNA-positive staining, and **P** Ki67-positive staining (*n* = 8 per group). Time-restricted feeding, TRF. Data were analyzed by area under the curve (AUC), two-tailed Student’s *t* test or two-way ANOVA with Tukey’s post hoc test. Error bars, when present, show the SEM. *P < 0.05; **P < 0.01; ***P < 0.001; ****P < 0.0001

To determine whether TRF also has antitumor activity in vivo, we subcutaneously injected 6 × 10^6^ A549 or 2 × 10^6^ H460 cells into BALB/c nude mice. Once the tumors became palpable, the mice were randomly assigned into a control ad libitum feeding (food accessible at any time) or TRF (food access restricted to 6 h during a nocturnal feeding time) group (Fig. 2A). Total food intake did not differ based on feeding protocol (Fig. 2B, C), whereas body weight was slightly but significantly lower in the TRF groups than in the corresponding control groups (Fig. 2D, E), suggesting that TRF may reprogram energy metabolism without imposing caloric restriction. TRF visibly attenuated A549 and H460 tumor volume in the xenograft mouse models (Fig. 2F). Tumor volume (Fig. 2G, H) and final tumor weight (Fig. 2I, J) were significantly lower in the TRF groups than in the corresponding control groups, while there were no significant differences in the weights of multiple organs or muscle (Fig. 2K, L), probably indicating no adverse effect upon TRF.

Because lung adenocarcinoma represents the most common histological subtypes of lung cancer (this contains the A549 cell line), we further evaluated the pathological biomarkers of tumor growth and toxicity in A549 xenograft tumors. Tumor sections from the TRF group showed a decrease in mitotic events (Fig. 2M, N), reductions in the percentages of PCNA-positive nuclei (Fig. 2M, O), and Ki67-positive (Fig. 2M, P) compared to those from the control group, indicating remarkable mitigation of tumor-associated pathological proliferation. Moreover, we performed pathological examinations of both lung and liver tissues to evaluate safety and found no significant pathological signs indicative of toxic effects in the TRF group relative to the control group (Additional file 1: Fig. S2). Collectively, the results reveal that TRF inhibits tumor growth in xenograft lung tumorigenesis mouse models without imposing caloric restriction.

### TRF suppresses lung tumorigenesis in urethane-administered mice

**Fig. 3:**
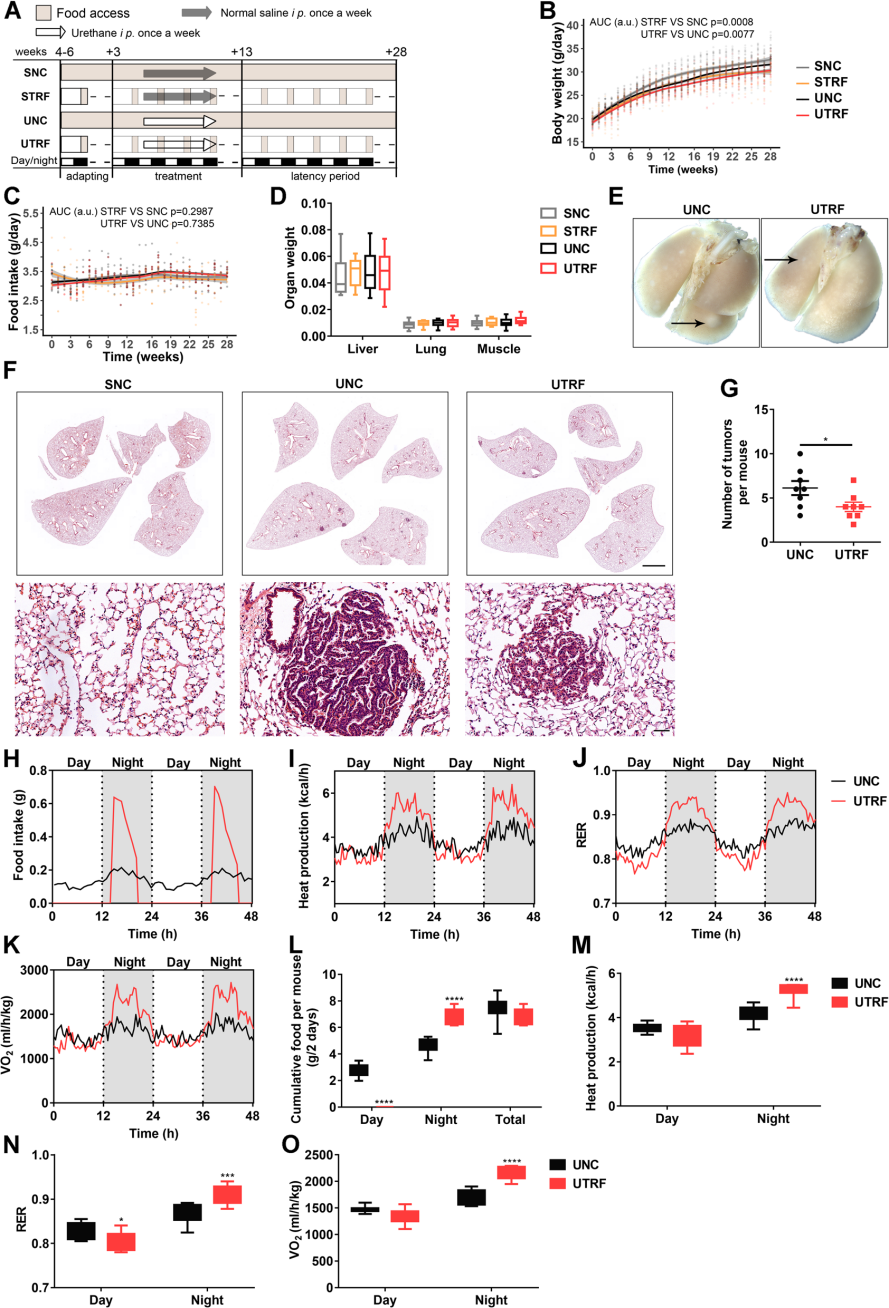
TRF suppresses lung tumorigenesis in a urethane-administered mouse model. **A** Schematic illustration of the experimental design for the urethane-administered mouse model. **B** Body weight trajectories (*n* = 16–26 mice per group). The dots on the graphs represent the values of individual mice. The gray outline to the lines represents the confidence interval. **C** Food intake trajectories (SNC: *n* = 20 mice; STRF: *n* = 16 mice; UNC: *n* = 26 mice; UTRF: *n* = 24 mice). **D** Organ weight (SNC: *n* = 16–20 mice; STRF: *n* = 15–16 mice; UNC: *n* = 18–26 mice; UTRF: *n* = 16–24 mice)**.** Organ mass values are expressed as the organ weight relative to total body weight. **E** Representative photographs of lung tumors. **F** H&E staining of whole-mount lungs showing reduced tumorigenesis following TRF in the top row and higher magnification images of H&E-stained lung tumors in the bottom row (top: scale bar = 2000 µm; bottom: scale bar = 50 µm). **G** Number of lung tumors at the end of the study (*n* = 8 mice per group). **H–O** Two 24-h energy metabolism cycles showing metabolic remodeling upon TRF (*n* = 8 mice per group, 24 weeks). The temporal patterns of **H** food intake, **I** heat production, **J** the respiratory exchange ratio (RER), and **K** oxygen consumption (VO_2_) is presented. Bar charts of **L** food intake, **M** heat production, **N** the RER, and **O** VO_2_ are shown. The data in **H–K** are shown as the mean. Data were analyzed by AUC, two-tailed Student’s *t* test, one-way ANOVA or two-way ANOVA with Tukey’s post hoc test. Saline-treated normal control, SNC; saline-treated time-restricted feeding, STRF; urethane-treated normal control, UNC; urethane-treated time-restricted feeding, UTRF. Error bars, when present, show the SEM. *P < 0.05; **P < 0.01; ***P < 0.001; ****P < 0.0001

To potentiate the prevention and treatment application of TRF in lung cancer patients, for whom lung cancer was commonly induced from chemical reagents exposure, we further determine whether the inhibitory effect of TRF on tumor growth is also observed in the chronic lung tumorigenesis model induced by chemical reagents of urethane. The C57BL/6 J mice in the control (fed an ad libitum control diet) and TRF groups (food access between ZT14 and ZT20) were randomized for treatment with saline or urethane by intraperitoneal injection every day for 10 consecutive weeks starting at 3 weeks control or TRF feeding, wherein the groups were as follows: saline-treated normal control (SNC), saline-treated TRF (STRF), urethane-treated normal control (UNC), and urethane-treated TRF (UTRF) (Fig. 3A). After a 15-week latency period, the experiment was ended (Fig. 3A). Analogous to the results in the xenograft mouse study, body weight was weakly but significantly decreased in the STRF and UTRF groups compared to the corresponding SNC and UNC groups, respectively, whereas food intake and organ weight relative to body weight showed no differences (Fig. 3B–D). The lung tumorigenesis model was successfully established, and the tumors in the UTRF group were smaller than those in the UNC group by gross examination (Fig. 3E) and histopathological examination (Fig. 3F). In addition, a gross pathological examination from this model further revealed that atypical adenomatous hyperplasia and papillary and solid adenoma were the two main pathological types (Fig. 3F), supported by our previous study [26]. Compared with UNC mice, UTRF mice had significantly fewer lung tumors (Fig. 3G), indicating that TRF also effectively impedes tumorigenesis in this chemically induced lung cancer model.

The above-described results indicated that TRF attenuates body weight without reducing caloric intake in mice, raising the question of whether TRF impacts energy metabolism in tumor-bearing mice. To address this, we measured energy expenditure in urethane-administered mice exposed to TRF or control feeding using indirect calorimetry. During the experiment, food consumption in the UTRF group occurred during the nocturnal phase, and total food consumption did not differ between the UTRF group and the UNC group (Fig. 3H, L). Heat production was significantly higher in the UTRF group than in the UNC group during the feeding phase (Fig. 3I, M), supporting the observation of weight loss in this study. Compared with that of the control group, the respiratory exchange ratio (RER) of the UTRF group showed a nocturnal increase, indicative of increased glycolysis during feeding, and a reduction during the daytime, reflective of an increase in fat oxidation during fasting (Fig. 3J, N). Additionally, oxygen consumption was increased at night in the UTRF group relative to the UNC group (Fig. 3K, O). Combined, TRF represses lung cancer tumorigenesis and progression, as well as rewires energy metabolism in urethane-administered mice.

### TRF reprograms the rhythms of metabolites and genes involved in glycolysis

Since TRF rewired energy metabolism in tumor mice, we next determined whether TRF impacts the metabolic factors and their rhythmicity in lung cancer. We first attempted to measure the change in serum glucose and lipids from serum samples being dynamically collected every 4 h during a 24-h period in xenograft lung tumorigenesis mouse models. The majority of serum measures, including glucose (GLU), low-density lipoprotein cholesterol (LDL-C), triglyceride (TG), insulin, and insulin-like growth factor 1 (IGF-1), did not show rhythmic levels in either the control or TRF group of A549 xenograft model mice; however, circadian rhythms were observed for high-density lipoprotein cholesterol (HDL-C) and total cholesterol (TCHO) in both groups (Additional file 1: Fig. S3 A-G and Table S1). Although GLU levels were decreased in the TRF group during the dark phase, the levels of many serum parameters were comparable between the TRF group and the control group (Additional file 1: Fig. S3A-G). HDL-C, LDL-C, TCHO, and TG were further evaluated in the urethane-induced lung tumorigenesis model from serum samples being dynamically collected every 6 h during a 24-h period and rhythms were analyzed (Additional file 1: Table S2). HDL-C showed a cyclical pattern only in the SNC and UTRF groups (Additional file 1: Fig. S3H). Moreover, HDL-C levels were decreased in saline-administered mice exposed to TRF (STRF group) compared to those on a normal diet (SNC group), but this decrease was not observed in urethane-treated mice (Additional file 1: Fig. S3H). LDL-C showed a cyclical pattern only in the UNC and UTRF groups and its levels showed no statistical difference in all compared groups (Additional file 1: Fig. S3I). TCHO was circadian merely in the SNC group, with its levels being significantly reduced under UTRF conditions compared to UNC conditions during the light phase (Additional file 1: Fig. S3J). TG was acyclic among the four groups and its level was not changed (Additional file 1: Fig. S3K). These findings suggest that TRF has a minor impact on the levels of these serum metabolic risk factors and their rhythmic regulation in lung cancer models, indicating that alternative metabolic factors responsible for the antitumor activity of TRF.

**Fig. 4:**
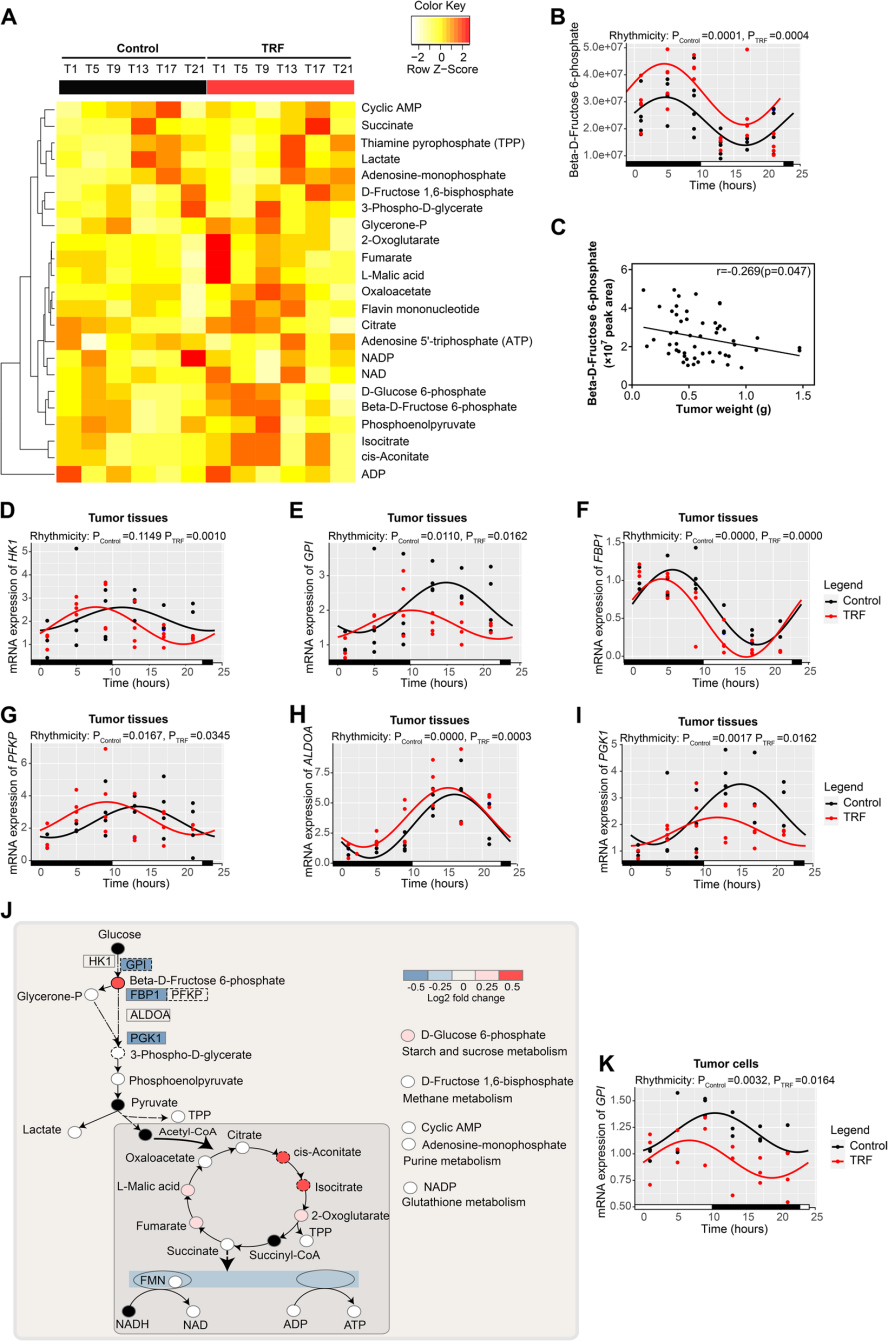
TRF reshapes the rhythms of metabolites and genes implicated in glycolysis. **A** Heatmap showing reprogramming of energy metabolites in tumor tissues of mice inoculated with A549 tumor cells upon TRF versus control treatment. **B** Abundance of beta-d-fructose 6-phosphate in tumor tissues from the mouse xenograft model collected at different times (ZT1, 5, 9, 13, 17, 21) over 24 h (Control: *n* = 5–6 mice per timepoint; TRF: *n* = 4–5 mice per timepoint). ZT0 indicates 10 pm, which was the start of the TRF intervention during the experiment. **C** Correlation analysis between beta-d-fructose 6-phosphate level and tumor weight (*n* = 55 mice). **D–I** qRT–PCR detects mRNA expression of rate-limiting enzymes implicated in beta-d-fructose 6-phosphate metabolism including **D** *HK1*, **E** *GPI*, **F** *FBP1*, **G** *PFKP*, **H** *ALDOA*, and **I** *PGK1*, in tumor tissues at different times over 24 h (Control: *n* = 3–4 mice per timepoint; TRF: *n* = 3–4 mice per timepoint). **J** The abundance of metabolites and genes involved in energy metabolism between the TRF and control groups. Log_2_-fold changes in genes and metabolites are color-coded; red represents an increase and blue represents a decrease upon TRF compared to control. Black dots represent metabolites not detected by MS. Only enzymes detected by qRT‒PCR are shown. **K** mRNA expression of *GPI* gene in A549 tumor cells in vitro was measured by qRT‒PCR at different times over 24 h (*n* = 3). ZT0 indicates the end of the two cycles of 24-h TRF intervention. Data were analyzed and visualized with circacompare or by Pearson correlation analysis. Error bars, when present, show the SEM

Metabolic reprogramming in cancer is characterized by the Warburg effect, which stipulates that most cancer cells rely on aerobic glycolysis to generate the energy required for cellular processes [29]. Therefore, we next applied targeted metabolomics to determine the temporal levels of metabolites involved in glycolysis and energy metabolism in tumor tissues from the A549 xenograft mouse model. Heatmap revealed that the control and the TRF groups exhibited opposite metabolites abundance tendencies (Fig. 4A). Rhythms were analyzed by circacompare algorithm and the parameters of rhythmicity, MESOR (rhythmically adjusted mean level of a response variable around which a wave function oscillates), amplitude, and phase were outputted (Additional file 1: Table S3). A total of 23 metabolites were identified and detected, of which 12 in the control group and 17 in the TRF group showed robust circadian rhythms; 10 of these metabolites showed rhythmicity under both conditions (Additional file 1: Fig. S4A). Notably, TRF did not reduce the lactate level, a primary measure of the Warburg effect, although lactate showed circadian rhythmicity in both groups (Additional file 1: Fig. S4A). While there were no significant differences in the amplitudes of these oscillating metabolites in both groups, the MESOR value of some metabolites, including beta-d-fructose 6-phosphate, fumarate, d-fructose 6-bisphosphate, isocitrate, and cis-aconitate, were consistently increased in the TRF group (Fig. 4B and Additional file 1: Fig. S4A-E). Additionally, the phases of isocitrate and cis-aconitate were shifted in the TRF group (Additional file 1: Fig. S4A, D, E). Correlation analysis suggested that among these MESOR-differential and phase-differential metabolites, only beta-d-fructose 6-phosphate, a metabolite enriched in glycolysis, showed a significantly negative correlation with tumor weight (Fig. 4C and Additional file 1: Fig. S4F, G). Combined, TRF reprogrammed the temporal levels of metabolites in tumor tissues compared with control treatment.

As the accumulation of beta-d-fructose 6-phosphate metabolite may be a result of enzyme suppression, several key rate-limiting enzymes implicated in beta-d-fructose 6-phosphate metabolism were examined by qRT-PCR. In tumor tissues, mRNA expressions of hexokinase 1 (*HK1)*, glucose-6-phosphate isomerase (*GPI)*, fructose-bisphosphatase 1 (*FBP1)*, phosphofructokinase, platelet (*PFKP)*, aldolase, fructose-bisphosphate A (*ALDOA)*, and phosphoglycerate kinase 1 (*PGK1)* all showed robust circadian rhythms in the control and TRF groups, except that mRNA expressions of HK1 showed acyclic expression in the control group (Fig. 4D–I). Three genes (*GPI*, *FBPI*, and *PGK1*) of them showed a downregulated mRNA expression and two genes (*GPI* and *PFKP*) of them showed a shifted phases in mRNA expression in the TRF group compared with the control group (Fig. 4D–I and Additional file 1: Table S4), suggesting the suppression of glycolysis (illustrated in Fig. 4J). Combined, the above results found that only the mRNA expression of *GPI* was downregulated meanwhile its phase was also shifted upon TRF, indicating a possible key role upon TRF. Therefore, we further evaluated the *GPI* mRNA expression pattern in A549 tumor cells by qRT-PCR and found that circadian *GPI* expression was consistent with that in tumor tissues (Fig. 4K). Moreover, compared to the control, TRF evoked the same change in GPI protein levels in A549 cells as in tumor tissues (Additional file 1: Fig. S5A). However, evaluation of the proliferation and migration assays of stable GPI-overexpressing and GPI-knockout cell lines showed that the relative colony area, relative cell proliferation, and relative cell migration were not significantly changed compared to the corresponding vector cell lines upon TRF, indicating that GPI does not impact the TRF-mediated antitumor effect (Additional file 1: Fig. S5B-H). These results indicate that while TRF reshapes glycolysis, this effect is not implicated in the antitumor activity of TRF, suggesting the involvement of alternative pathways.

### TRF shapes the rhythms of clock genes in a cell-or tissue-specific manner

Aside from glycolysis, circadian rhythm disruption is emerging as another characteristic of tumor [3]; therefore, we next explored whether circadian rhythm genes were key factors in anti-tumor activity of TRF. Because lung adenocarcinoma is the main pathological type of lung cancer [30], we focused on the molecular mechanism by which TRF limited lung adenocarcinoma. To identify key clock genes that are likely involved in TRF-mediated tumor suppression, we first screened clock genes and regulators related to lung adenocarcinoma using public datasets (Additional file 2). In both the TCGA and GEO datasets, the candidate clock genes cryptochrome circadian regulator 1 (*CRY1),* cryptochrome circadian regulator 2 *(CRY2),* F-box and leucine rich repeat protein 3 *(FBXL3),* timeless circadian clock *(TIM)*, and G protein-coupled estrogen receptor 1 (*GPER1)* were identified based on their consistently lower or higher expression levels in lung adenocarcinoma tissues than in normal tissues (Additional file 1: Fig. S6A-C). Survival analysis further revealed that *CRY1, CRY2, GPER1,* and *FBXL3* were negatively related to survival of lung adenocarcinoma patients, while *TIM* were positively related to survival (Additional file 1: Fig. S6D).

**Fig. 5:**
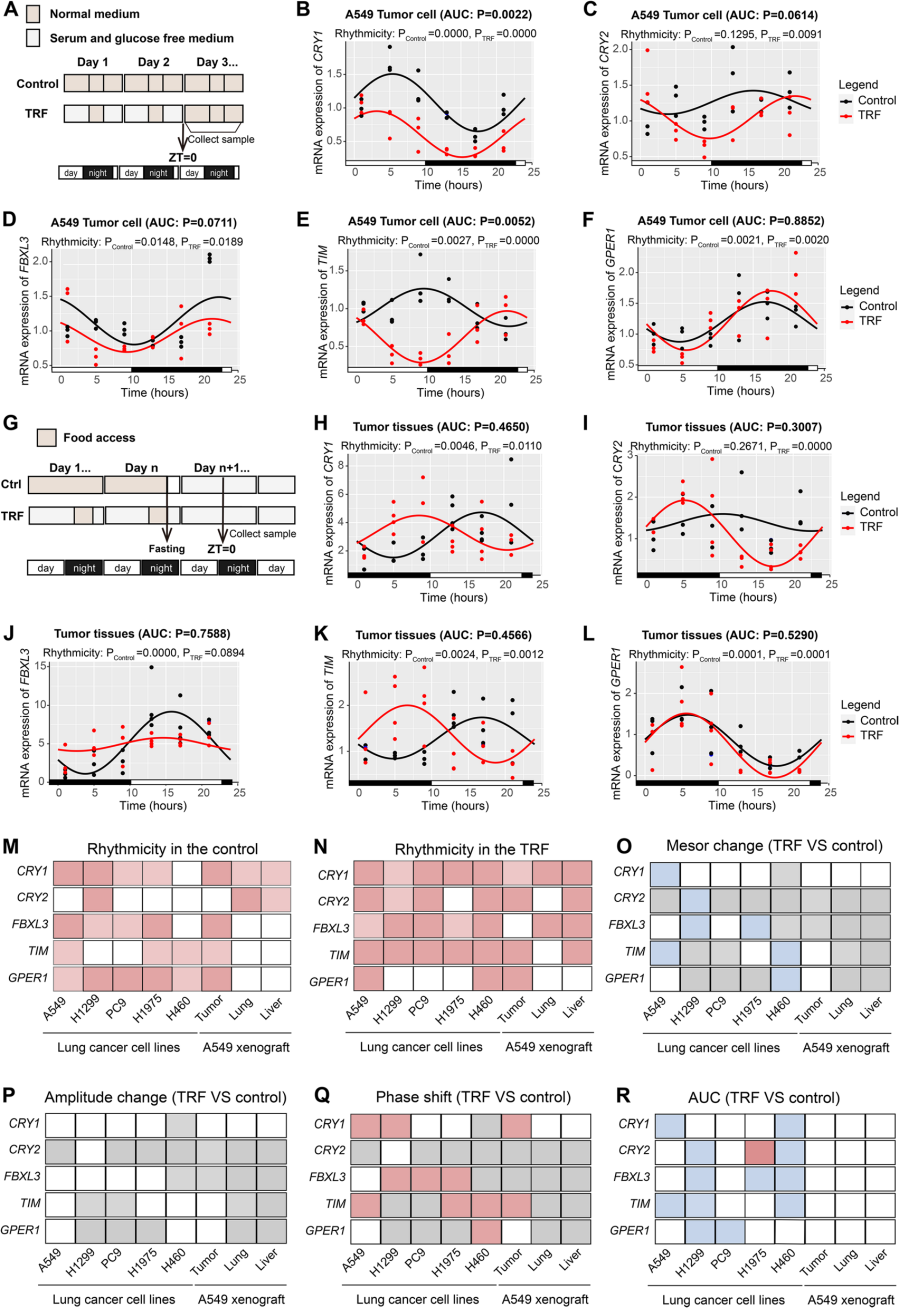
TRF remodels the rhythmic expression of clock genes in vitro and in vivo. **A** Schematic illustration of in vitro sample collection. **B–F** mRNA expression in A549 tumor cells at different times (ZT1, 5, 9, 13, 17, 21); the genes included **B** *CRY1,* **C** *CRY2*, **D** *FBXL3*, **E** *TIM*, and **F** *GPER1* (*n* = 3 per timepoint in each group). ZT0 indicates the end of the two cycles of 24-h TRF intervention. **G** Illustration of the sample collection for A549 xenograft-bearing mice. **H–L** Gene expression in tumor tissue at different times; the genes included **H** *CRY1,* **I** *CRY2*, **J** *FBXL3*, **K** *TIM*, and **L** *GPER1* (*n* = 3–4 per timepoint in each group). ZT0 indicates 10 pm, which was the start of the TRF intervention in mice. **M–R** Visual plots showing rhythmic and AUC results for clock genes from different cell lines and A549 xenograft model tissues analyzed with circacompare package. **M, N** Rhythmicity in the **M** control and **N** TRF groups. Dark and light red both indicate rhythmicity in the control or TRF groups (*P* value < 0.05); light red indicates higher *P* value upon TRF versus control. **O** MESOR. Blue indicates that MESOR values were decreased upon TRF versus control, light gray identifies genes that could not be compared since there was no circadian oscillation in both groups, and white indicates nonsignificant results. **P** Amplitude upon TRF versus control. Light gray identifies genes that could not be compared, and white indicates nonsignificant results. **Q** Phase-shift upon TRF versus control. Red indicates *P* value < 0.05, light gray identifies genes that could not be compared, and white indicates nonsignificant results. **R** AUC analysis. Red and blue indicates higher and lower AUC values upon TRF versus control, respectively; white indicates nonsignificant results. Error bars, when present, show the SEM

We then examined the temporal expression of the five clock genes in four distinct human lung adenocarcinoma cell lines and one human large cell lung cancer cell line at 4-h intervals over a 24-h period (ZT1, 5, 9, 13, 17, and 21), and analyzed their rhythmicity by circacompare (Fig. 5A–F, M, N, and Additional file 1: Table S5, 6). The mRNA expression levels of *CRY1*, *CRY2*, *FBXL3*, *TIM*, and *GPER1* showed cell line-specific circadian rhythms in either the control or TRF group for the majority of the cell lines (Fig. 5B-F, M, N and Additional file 1: Fig. S7). However, of these five genes, only the circadian expression of the *TIM* gene was enhanced or established in the TRF group compared to the control group for each cell line (Fig. 5B–F, M, N and Additional file 1: Fig. S7). *CRY1*, *CRY2*, *FBXL3*, *TIM*, and *GPER1* expressions were frequently lower in the TRF group than in the control group (Fig. 5B–F, O, R and Additional file 1: Fig. S7). Although there were no differences in the amplitudes of the 5 dual-oscillating genes (Fig. 5P), the phases of *CRY1*, *FBXL3*, and *TIM* gene expression were significantly shifted in certain cell lines (Fig. 5Q). Altogether, these data suggest that TRF induces cell-specific programming of some circadian oscillations in vitro, but conservatively rewires *TIM* rhythm.

To further investigate whether TRF modulated circadian oscillations in peripheral tissues in vivo, we next examined mRNA expressions of clock genes in the liver, lung, and tumor tissues by collecting samples from A549 xenograft mice under TRF conditions every 4 h (Fig. 5G and Additional file 1: Tables S5, S7). In the liver tissue, the mRNA expression of *Cry1* and *Cry2* showed circadian rhythms in both the TRF group and the control group, but there were no significant differences between groups in the MESOR, amplitude, or phase; meanwhile, *Gper1* expression was arrhythmic in the two groups (Fig. 5M–Q and Additional file 1: Fig. S8A, B, E). Rhythms in *Fbxl3* and *Tim* expression were restored by TRF, but the expression levels of these genes were not significantly different in the TRF and control groups (Fig. 5M, N, R and Additional file 1: Fig. S8C, D). In the lung tissue, *Cry1, Cry2*, and *Fbxl3* expression showed significant periodicity in both the control group and the TRF group, but *Tim* and *Gper1* levels were acyclic (Fig. 5M, N and Additional file 1: Fig. S8F-J). Analogous to the liver tissue results, the expression levels of these genes in the lung tissue did not differ between the two groups (Fig. 5O and Additional file 1: Fig. S8F-J). Overall, this lung and liver data indicate that TRF rewired rhythms of clock genes in nontumor tissues but did not affect the expression level.

In the tumor tissue, *CRY1, TIM,* and *GPER1* expressions showed circadian rhythms in both the control and the TRF groups, but the circadian rhythm of *TIM* expression was more robust in the TRF group, while *CRY2* were acyclic in the control group and *FBXL3* were acyclic in the TRF group (Fig. 5H–L, M, N). Similarly, no changes in the MESOR or amplitude were observed for any of the dual-oscillating genes (Fig. 5H–L, O, P). Of these five genes, only *CRY1* and *TIM* genes expressions showed a significant phase shift in the TRF group compared to the control group (Fig. 5H–L, Q), consistent with our results for the A549, H1975, and H460 cell lines (Fig. 5Q). Moreover, although the average *CRY1*, *CRY2*, and *TIM* gene expression levels in tumor tissue did not differ between the TRF and control groups, the levels during the day phase were significantly lower in the TRF group (Fig. 5H, I, K, R), in agreement with the A549 cell results (Fig. 5R). Thus, taken together, these results suggest that TRF remodels circadian oscillations in a cell- or tissue-specific manner. However, more importantly, after TRF intervention, only *TIM* gene in these five genes was found to consistently align rhythm of tumor cells to that of tumor tissues, including a decrease in expression during the day phase (Fig. 5E, K), consistent with the circadian rhythm driven by the intermittent TRF dietary regimen [31]. Further, previous study reported that TIM enhanced proliferation and migration in lung adenocarcinoma cells [24], suggesting an important role in lung cancer. Therefore, we speculated that TIM regulation may play a vital role in tumor suppression by TRF.

### TIM contributes to TRF-dependent changes in tumor suppression

**Fig. 6:**
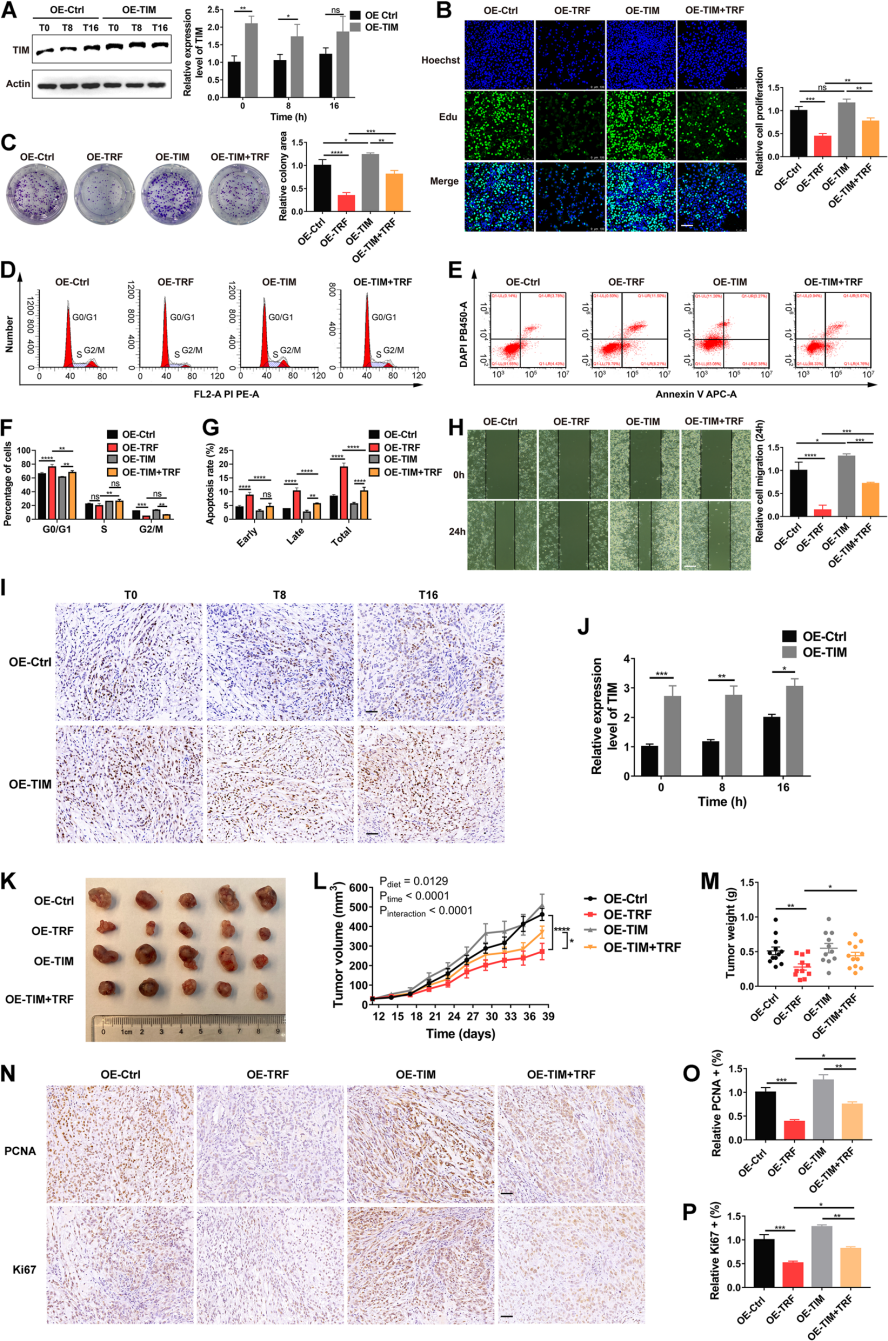
TIM contributes to TRF-dependent changes in tumor suppression in vitro and in vivo. **A** The protein expression of TIM in stable TIM-overexpressing A549 cells. Left: representative blots; right: quantitative results. Expression relative to OE-control group at T0. **B** EdU proliferation assay in stable TIM-overexpressing A549 cells. Left: representative images (scale bar = 100 µm); right: quantitative results (*n* = 3). **C** Colony formation proliferation assay. Left: representative images; right: quantitative results (*n* = 3). **D, E** Representative images of a flow cytometry assay to detect **D** the cell cycle (PI staining) and **E** cell apoptosis (Annexin V staining for early apoptosis and DAPI staining for late apoptosis) in stable TIM-overexpressing A549 cells. **F, G** Quantification of the **F** cell cycle and **G** cell apoptosis (*n* = 3). **H** Wound healing migration assay. Left: representative images (scale bar = 100 µm); right: quantitative results (*n* = 3). **I, J** TIM expression of the xenograft model mice inoculated with stable TIM-overexpressing A549 cells detected by immunohistochemistry; **I** representative images (scale bar = 50 µm) and **J** quantification result (*n* = 3). **K** Photograph of dissected tumors from xenograft model mice injected with TIM-overexpressing A549 cells with or without TRF. **L** Tumor volume (*n* = 11–12 mice per group). **M** Tumor weight (*n* = 11–12 mice per group). **N**–**P** Histopathology. **N** Representative PCNA and Ki67 staining of tumor tissue (scale bar = 50 µm). **O, P** Quantification of the **O** PCNA-positive staining and **P** Ki67-positive staining (*n* = 8 in each group). OE-Ctrl, negative-overexpression-control; OE-TRF, negative-overexpression-control with TRF; OE-TIM, TIM-overexpression; and OE-TIM + TRF, TIM-overexpression with TRF. Data were analyzed by one-way or two-way ANOVA with Tukey’s post hoc test. Data was expressed as mean ± SD for Fig. 6A–H and mean ± SEM for Fig. 6 J–P. *P < 0.05; **P < 0.01; ***P < 0.001; ****P < 0.0001

To ascertain whether TIM is involved in tumor suppression induced by TRF, we constructed an overexpressed A549 cell line with altered average TIM expression levels using lentivirus-mediated overexpression (Fig. 6A). TIM overexpression promoted cell proliferation in TRF-treated cells, but the proliferation ability was still lower compared to control-treated cells, suggesting that TIM overexpression partially attenuated the inhibitory effect of TRF on cell proliferation (Fig. 6B). Moreover, TIM overexpression enhanced the colony-forming ability of TRF- and control-treated cells, but the fold increase in proliferation was greater in the TRF group than in the control group (Fig. 6C). Likewise, TIM overexpression partially reversed the TRF-mediated induction of cell cycle arrest in G0/G1 and apoptosis (Fig. 6D–G). As further indicated by a wound healing assay, TIM overexpression partially attenuated the suppression effect of TRF on cell migration, which was similar to those for proliferation assays (Fig. 6H), demonstrating that downregulated TIM partially contributes to the TRF-mediated inhibition of tumor cell proliferation and migration.

Finally, to examine whether this inhibitory effect of TRF on tumors is mediated by TIM in vivo, a mouse xenograft model was established by implanting 1 × 10^7^ stable TIM-overexpressing A549 cells into the back subcutaneous tissue of nude mice, and tumor tissues were harvested at the T0, T8, and T16 time points. TIM protein expression in tumor tissues was higher in mice bearing TIM-overexpressing tumors than in those bearing control tumors at the T0, T8, and T16 time points, but expression in the TIM-overexpressing group was comparable at different time points (Fig. 6I, J). The tumor growth inhibition induced by TRF was partially reversed by TIM overexpression (Fig. 6K), as illustrated by reductions in final tumor weight and volume (Fig. 6L, M). Likewise, a reduction of in the percentages of PCNA-positive and Ki67-positive induced by TRF was partially reversed by TIM overexpression (Fig. 2N, P). Taken together, these results demonstrate that TIM is involved in TRF-mediated tumor growth inhibition in vitro and in vivo.

### Autophagy regulation by TIM is involved in TRF-mediated tumor suppression

**Fig. 7:**
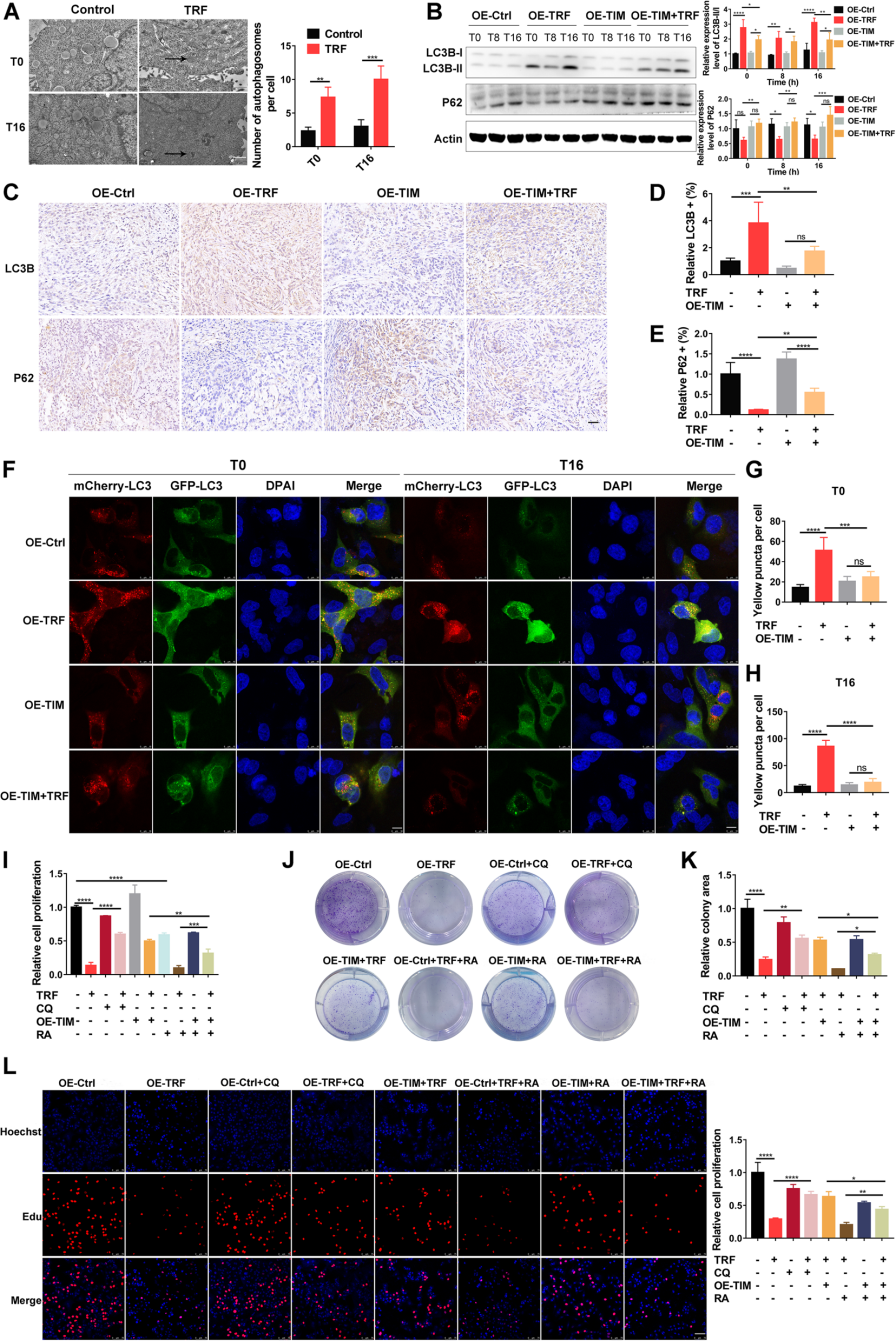
TIM overexpression attenuates the tumor-suppressive effect of TRF by regulating autophagy. Transmission electron microscopy shows autophagosomes in TRF-treated A549 cells. Left: representative images (scale bar = 1 µm); right: quantitative analysis (*n* = 3). Arrows indicate the autophagosomes. Autophagosomes were counted in 20 cells in three independent replicates. **B** The protein levels of LC3B and P62 in stable TIM-overexpressing A549 cells, left: representative blots; right: quantitative analysis (*n* = 3). ZT0 indicates the end of the two cycles of 24-h TRF intervention. **C** Immunohistochemistry. Representative LC3B and P62 staining of tumor tissue from A549 xenograft model mice injected with stable TIM-overexpressing cells (scale bar = 50 µm). **D** Quantification of LC3B-positive staining (*n* = 5 in each group). **E** Quantification of P62-positive staining (*n* = 5 in each group). **F**–**H** Autophagic flux assays. Stable TIM-overexpressing A549 cells were transfected with mCherry-GFP-LC3 plasmids. **F** Representative images (scale bar = 10 µm). **G, H** Quantitative analysis of autophagosomes (mCherry + /GFP +) based on yellow puncta per cell at **G** T0 and **H** T16 (*n* = 3 in each group). **I** CCK-8 assay in stable TIM-overexpressing A549 cells after culture with 15 nM rapamycin (RA) for 12 h or 30 µM chloroquine (CQ) for 24 h with or without TRF (*n* = 3–4 in each group). **J**,** K** Colony formation assay in TIM-overexpressing cells after culture with RA or CQ upon TRF; **J** representative images, and **K** quantitative analysis (*n* = 3 in each group). **L** EdU assay. Left: representative images (scale bar = 75 µm); right: quantitative analysis (*n* = 3). OE-Ctrl, negative-overexpression-control; OE-TRF, negative-overexpression-control with TRF; OE-TIM, TIM-overexpression. Data were analyzed by one-way or two-way ANOVA with Tukey’s post hoc test. Error bars, when present, show the SD. *P < 0.05; **P < 0.01; ***P < 0.001; ****P < 0.0001

TRF has been reported to regulate autophagy [31, 32], which is widely related to tumor growth [33, 34]; therefore, we speculated that autophagy may be also involved in TRF-mediated tumor suppression. Further, if this mechanistic hypothesis were to hold true, we speculated that a possible regulatory relationship appears to exist of TIM and autophagy. To test this conjecture, we first evaluated the degree of autophagy in A549 tumor cell by electron microscopy and found increased formation of autophagosomes upon TRF compared to control treatment at both the T0 and T16 time points (Fig. 7A). The ratio LC3B-II/I protein (autophagy marker) were increased, while those of P62 (substrate receptor degraded by autophagy) were decreased in the TRF group compared to the control group at the T0, T8, and T16 time points; however, these changes in protein levels were partially or fully reversed when TIM was overexpressed at either T0, T8, or T16 time points (Fig. 7B). Immunohistochemistry of tumor tissues prepared from TIM overexpressing mouse xenograft tumor models further confirmed these findings, which showed that TIM overexpression attenuated autophagic activity induced by TRF (Fig. 7C–E). Additionally, autophagic flux assays were conducted by monitoring the fusion protein mCherry-GFP-LC3 under confocal laser scanning microscope; in the assays, autophagosomes express both mCherry and GFP, displaying yellow in merged images. TRF caused the accumulation of autophagosomes, but this accumulation was reduced nearly to the level of control group (OE-Ctrl) in stable TIM-overexpressing cells at the T0 and T16 time points (Fig. 7F–H). These results suggest that TIM mitigates the autophagy induced by TRF.

To clarify whether TRF suppresses tumors by upregulating autophagy via the regulation of TIM, we next examined the proliferation of control or stable TIM-overexpressing A549 cells with or without TRF in combination with the known autophagy inducer rapamycin (RA) or autophagy inhibitor chloroquine (CQ). CCK8, colony formation, and EdU assays showed that CQ partially reversed the TRF-induced suppression of cell proliferation (Fig. 7I–L). Moreover, in the same assays, rapamycin treatment partially reversed the pro-proliferative effect of stable TIM overexpression in cells exposed to TRF (Fig. 7I-L). These results, along with those described above, suggest that TRF decreases TIM expression, which leads to an increase in autophagy that consequently suppresses tumor growth.

### Combining TRF and TIM inhibition enhances antitumor activity

**Fig. 8:**
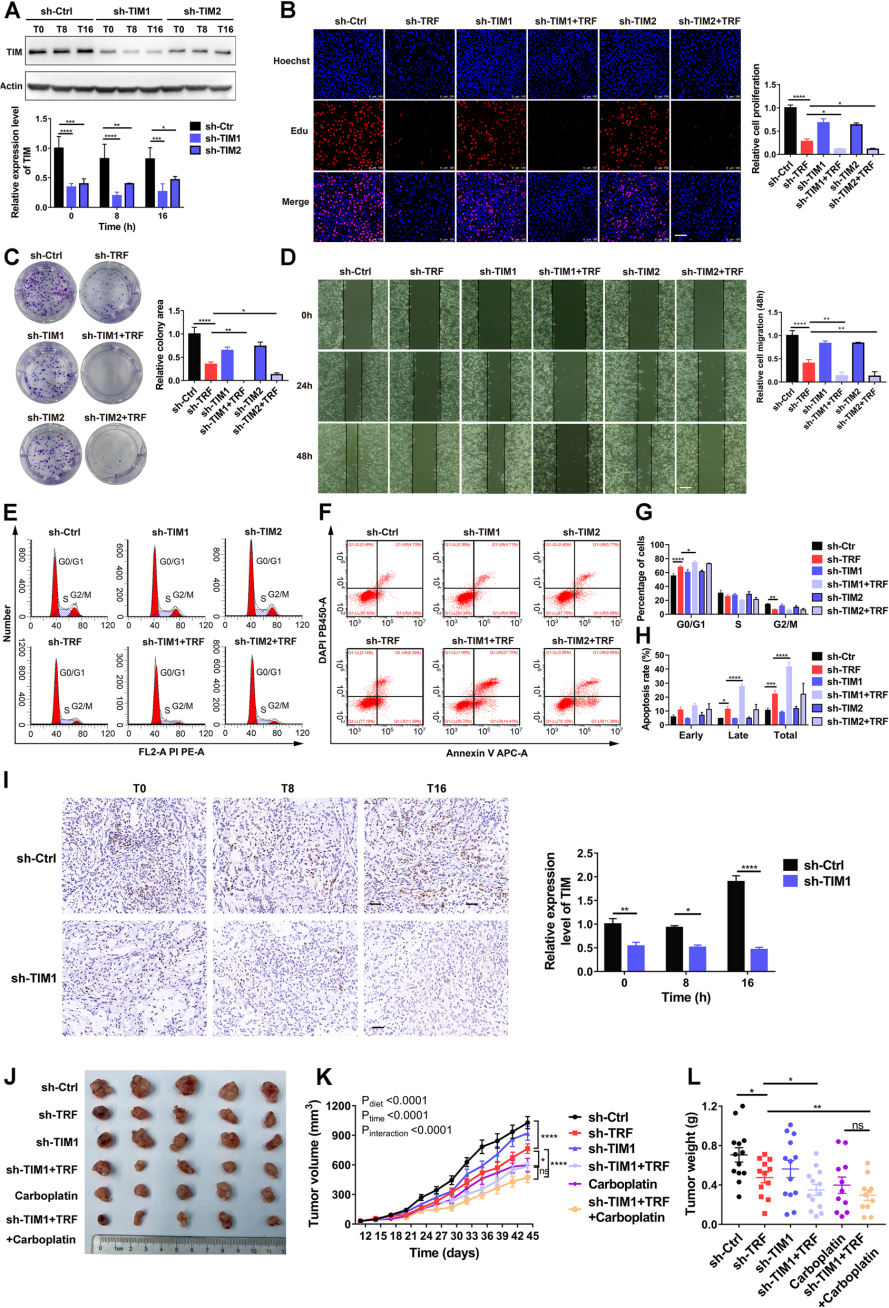
Combining TIM inhibition with TRF enhances antineoplastic efficacy in lung cancer. **A** TIM protein expression in A549 cells was robustly inhibited by a lentivirus expressing TIM-targeting shRNA (sh-TIM1 and sh-TIM2) versus control shRNA (sh-Ctrl) at ZT0, ZT8, and ZT16. ZT0 indicates the end of the two cycles of 24-h TRF intervention. Top: representative blots; bottom: quantitative results. **B** EdU proliferation assay in two stable TIM-knockdown A549 cell lines. Left: representative images (scale bar = 100 µm); right: quantitative results (*n* = 3). **C** Colony formation assay. Left: representative images; right: quantitative results (*n* = 3). **D** Wound healing assay. Left: representative blots (scale bar = 100 µm); right: quantitative results (*n* = 3–4). **E**–**F** Representative images of a flow cytometry assay to evaluate the **E** cell cycle and **F** cell apoptosis of stably transfected TIM-knockout A549 cells. **G, H** Quantification analysis of the **G** cell cycle and **H** cell apoptosis data (*n* = 3). **I** TIM expression from ZT0, ZT8, and ZT16 in the xenograft mouse model inoculated with stable sh-TIM1 A549 cells was confirmed by immunohistochemistry. Left: representative images (scale bar = 50 µm); right: Quantification of TIM expression (*n* = 3). All expressions are relative to sh-Ctrl: T0. ZT0 indicates 10 pm, which was the start of the TRF intervention in mice. **J** Photograph of tumors formed by implantation of stable sh-TIM1 A549 cells. **K** Tumor volume (*n* = 11–13 mice per group). **L** Tumor weight (*n* = 11–13 mice per group). Data were analyzed by one-way or two-way ANOVA with Tukey’s post hoc test. sh-Ctrl, negative shRNA control; sh-TRF negative shRNA with TRF; sh-TIM1, TIM shRNA1; sh-TIM2, TIM shRNA2. Data was expressed as mean ± SD for Fig. 8**A**-**H** and mean ± SEM for Fig. 8**I**, **K**, **L**. *P < 0.05; **P < 0.01; ***P < 0.001; ****P < 0.0001

Considering the vital roles of TIM in the antitumor effects of TRF, we further hypothesized that combined TIM inhibition and TRF might have a synergistic antineoplastic effect. To test this hypothesis, A549 cells were stably transfected with TIM-specific shRNAs (sh-TIM1 or sh-TIM2) or negative control shRNA (sh-Ctrl). The efficiency of TIM knockdown was confirmed by western blotting (Fig. 8A). EdU and colony formation assays showed that TIM knockdown led to more robust suppression of tumor cell proliferation upon TRF than did sh-Ctrl treatment (Fig. 8B, C). The TRF-induced suppression of cell migration was further enhanced in TIM-knockdown A549 cells relative to sh-Ctrl-infected cells (Fig. 8D), indicating that combining TRF with TIM inhibition enhanced antitumor activity. This synergistic antineoplastic effect was further confirmed by flow cytometry assays that showed increased cell cycle arrest and apoptosis upon TRF in the two stable TIM-knockdown A549 cell lines (Fig. 8E–H).

We further evaluated the efficacy of combination treatment with TRF, TIM inhibition, and chemotherapy in vivo; in this experiment, mice bearing sh-Ctrl or sh-TIM1 A549 xenografts administered a TRF or control diet were treated with or without carboplatin. Sh-TIM1 induced efficient TIM knockdown in tumor tissues compared with sh-Ctrl (Fig. 8I). In agreement with the in vitro proliferation and apoptosis results, mice treated with TRF had significantly decreased tumor volume and weight compared to mice given control chow (sh-Ctrl vs. sh-TRF) (Fig. 8J–L). TIM knockdown significantly enhanced the effect of TRF on tumor inhibition (sh-TRF vs. sh-TIM1 + TRF), which was comparable to the effect of carboplatin alone (carboplatin vs. sh-TIM1 + TRF) (Fig. 8J–L). Strikingly, the combination of TRF, TIM1 knockdown, and carboplatin also inhibited tumor growth (sh-TRF vs. sh-TIM1 + TRF + carboplatin), with this treatment displaying the most robust antitumor efficacy tendency (Fig. 8J–L). Overall, these data suggest that targeting TIM is a strategy with high potential to augment the antitumor efficacy of treatment with TRF.

## Discussion

Accumulating evidence has suggested an oncogenic effect of diurnal disruption on cancer progression [3–5, 35, 36]. To test whether targeting and reprogramming circadian rhythm by dietary strategy suppressed lung cancer progression, we adopted time-restricted feeding (TRF) paradigm to elucidate whether and how 6-h TRF impacts lung cancer progression and rewires circadian metabolism. To our knowledge, using cell lines, xenograft mice, and chemical-treated mice models, this study is the first to demonstrate that compared to normal feeding, 6-h TRF inhibits the initiation and progression of lung cancer and reprograms the circadian rhythm of clock genes and glycolytic metabolism. In vitro and in vivo experiments demonstrated that TRF activated autophagy by downregulating TIM, resulting in tumor suppression. More broadly, combined TRF and TIM inhibition enhances the anti-tumor efficacy of TRF alone in a lung adenocarcinoma xenograft model.

In recent years, accumulating evidence has shown that, in addition to the quantity and quality of food, the time of feeding is crucial for the well-being of the organism; this concept is termed “chrono-nutrition” and is embodied by the typical feeding paradigm of TRF [37–39]. Considerable research has demonstrated that TRF substantially benefits health, ranging from reducing body weight and visceral fat mass to improving metabolic risk measures and extending lifespan [31, 40–42]. Although data are limited to a few animal studies, TRF intervention has been shown to have an antineoplastic effect in various cancers [15–17, 20]. Animal or population evidences of TRF effect on lung cancer are however arising from only two experiments [19, 43]. Both studies have found that 12-h TRF abrogated the obesity-enhanced spontaneous metastasis of Lewis lung cancer, as well as restored the health phenotypic impairment or shifted the plasma metabolic profiles changes driven by high-fat diet [19, 43]. Although the above studies have both demonstrated the potential inhibitory effects of 12-h TRF on lung cancer metastasis within the context of high-fat diet-induced obesity, the impact of TRF on lung cancer growth and progression in nonobese animals remains largely unknown. Building on these prior studies, our work demonstrates that 6-h TRF inhibited the initiation and progression of lung cancer in nonobese animals. Compared with the control group, tumor volume and tumor weight were visibly attenuated in the TRF group in the xenograft mouse and chemical induced lung cancer models. Considering cancer patients are commonly non-obese, thus TRF may be a promising nutrient therapy against lung cancer. However, whether there is a difference in the antitumor effect of TRF between obese and nonobese individuals remains largely unknown and requires further investigation.

In this study, we also found that 6 h-TRF reduced body weight by increasing energy metabolism without reducing caloric intake, consistent with the results of previous studies showing that TRF enhances overall oxidative metabolism and reduces body weight [44]. We next determined whether TRF impacts the metabolic factors and their rhythmicity in lung cancer. Partially inconsistent with previous studies [15, 44], although GLU levels were decreased in the TRF group during the dark phase, the levels of many serum parameters were comparable between the TRF group and the control group, which may be due to the background difference between normal and obesogenic diets. Using targeted metabolomic and qRT‒PCR experiments, we further found that 6-h TRF suppressed the temporal levels of energy metabolites and glycolytic enzymes in tumor tissues from a mouse xenograft model. Moreover, the rhythms of these metabolites and enzymes in tumors were enhanced by 6-h TRF. These findings are supported by previous studies showing TRF improves the disordered metabolic rhythm induced by a high-fat diet or obesity [45]. Many studies have demonstrated that targeting glycolytic metabolism is crucial for inhibiting tumor growth and identified the enzymes and metabolites related to glycolysis that are critical to cancer cells and widely involved in tumor growth, metastasis, and prognosis [46–48]. Similarly, we found glycolytic suppression overall; in particular, glycolytic gene GPI mRNA expression was persistently inhibited by TRF in tumor tissues and cells. This observation is consistent with previous studies showing that GPI overexpression aggravates the metastatic potential of non-small cell lung cancer and glioblastoma [47, 49]. However, we did not find GPI to be involved in TRF-mediated antitumor effects in vitro.

We found that in tumor contexts, TRF, in which feeding was restricted to 6 h in this study, reprogrammed the rhythmic expression of clock genes in a cell-specific (in vitro) or tissue-specific (in vivo) manner. These findings agree with those of previous studies. Nutrition is among the dominant external factors known to align the biological clock, and the concept of TRF was developed in the context of circadian rhythm, in which meal timing is coordinated with the body’s daily rhythms [8, 10]. Moreover, meal timing or TRF has also been shown to significantly improve circadian rhythms in distinct contexts, such as in cardiovascular disease, breast cancer, and pancreatic cancer [15, 16, 50]. Meal timing restricted from 9 am to 1 pm attenuates tumor growth driven by a high-fat diet in pancreatic adenocarcinoma by reinforcing the rhythmic expression of clock genes [16]. It is interesting to note that a different TRF regimen (night vs morning) and a high-fat diet seem to reach similar conclusions about the effect of TRF on cancer and clock genes. We speculate that the specific timing of TRF is not important to achieve antitumor effects and rewire rhythmic expression of clock genes. However, further study is warrant to investigate the impact of different TRF regimen on rhythms of clock genes and circadian metabolism.

More importantly, we found that in the context of TRF, the phase of the clock gene TIM was significantly shifted in tumors under most circumstances; this shift was paralleled by decreased TIM mRNA expression during the day phase, consistent with the rhythmic pattern driven by the intermittent TRF dietary regimen [51]. Notably, the effect of TRF on the average expression level of TIM was distinct in vitro and in vivo, which may be explained by the difference in applying TRF in different systems. TIM was highly expressed in lung cancer and cervical cancer and was significantly correlated with poor survival [25, 52]. TIM overexpression exhibited a protective effect on cancer cells against replication stress, independent of the checkpoint mechanism [53]. TIM depletion suppressed cell proliferation and enhanced cisplatin sensitivity in cervical cancer [54]. A recent study further found that TIM promoted the proliferation and migration of lung adenocarcinoma cells [24]. However, conversely, knockdown of TIM promoted breast cancer cell invasion and metastasis [55] and loss of TIM induced tumor progression in colorectal cancer [56]. Consistent with previous lung and cervical cancer studies, we found that TIM was overexpressed in lung cancer and TIM inhibition has a modest antitumor effect in lung cancer, but more robust evidence showed that TIM was partially involved in TRF-mediated tumor suppression. These results highlight the role of TIM in the antitumor effect of TRF in specific tumor type and further prompt us that TIM gene regulation at specific times appears to be of importance, which is in line with the concept of chronotherapy by adjusting the therapy time to adapt to the rhythm of the cancer [57]. However, further in-depth study for investigating the role of TIM gene regulation at specific times in TRF-mediated tumor suppression is warranted. The current study also demonstrated that combined TRF and TIM knockdown enhanced antitumor efficacy of TRF alone in A549 mouse xenografts, indicating that TRF in combination with TIM inhibition may be a promising strategy to potentiate standard cancer therapies. These results were supported by our previous finding demonstrating that a 6-h TRF enhanced the anti-tumor effects of cisplatin in cisplatin-resistant and cisplatin-sensitive lung cancer cells with TRF-mimicking paradigm [58].

We also found that autophagy regulation by TIM is involved in TRF-mediated tumor suppression. First, TIM overexpression partially reversed the accumulation of autophagosomes induced by TRF; this result was paralleled by decreased LC3B and increased P62 protein expression. In A549 cells, the autophagy inducer CQ partially reversed the TRF-mediated suppression of proliferation, further suggesting that TRF regulates autophagy. Moreover, treatment with the autophagy inducer rapamycin partially restored the TRF-mediated decrease in proliferation in the context of TIM overexpression, demonstrating that autophagy primarily contributes to the mechanism by which TRF regulates TIM to inhibit cancer. Previous studies have reported that autophagy plays a vital role in tumor growth [33, 34, 59]; however, autophagy frequently plays dual roles, mediating tumor suppression or tumor promotion in different contexts [33]. Specifically, in early tumorigenesis, autophagy is generally sufficient to suppress cancer growth and progression [33]. Therefore, we concluded that autophagy induced by TRF potentiates the observed antitumor effect. Consistently, autophagy induction by caloric restriction mimetics strengthens anticancer immunosurveillance [60], and fasting improves sensitivity of tumor cells to chemotherapeutic agents by upregulating autophagy flux [61]; both consistent with the beneficial role of autophagy involved. These data indicate that targeting autophagy is a promising interventional strategy for the treatment of cancer. However, how TIM regulates autophagy remains unknown and requires further study.

Although this study demonstrates that TRF significantly inhibits lung cancer growth, it has certain limitations. First, this experiment was performed with mice of only one sex (male). However, the mortality and morbidity of lung cancer in women have been increasing for many decades [1], so there may be the sex-specific differences in TRF-mediated anti-tumor effect. Second, although we observed that 6-h TRF had significant antitumor effects in lung cancer, we did not investigate the effects of other feeding windows, such as 4-h TRF, 8-h TRF, early TRF, or late TRF, on lung cancer. Because different time windows may have different effects on health [8], additional studies are needed to investigate different feeding windows for TRF to determine how to maximize tumor suppression in lung cancer. Third, in vivo experiment of autophagy was not performed in this study, which would strength our findings; however, our current results still strongly support the findings that autophagy regulation by TIM is involved in TRF-mediated tumor suppression. Fourth, although we used 6-h TRF-mimicking cellular medium, we struggled to completely recapitulate the in vivo TRF intervention, which could result in different metabolism response upon TRF. Finally, we explored the anti-tumor effect of TRF only in two xenograft mouse models and a chemical-treated mouse model, further study is required to demonstrate the anti-tumor effect of TRF in other more lung cancer models, such as Kras-driven genetically engineered mice models. Nevertheless, our study provides novel evidence that 6-h TRF without energy restriction effectively inhibits lung cancer growth and progression.

## Conclusion

In conclusion, we found for the first time that compared to normal ad libitum feeding, 6-h TRF inhibited lung cancer growth and progression, and reprogrammed the circadian rhythm. We also demonstrated that the anti-tumor effect upon TRF was partially mediated by the rhythmic downregulation of the TIM and the subsequent activation of autophagy. Combining TRF with TIM inhibition further enhanced the anti-tumor effect. Our results provide in vitro and in vivo evidence of TRF on lung cancer suppression and suggest that adjusting meal timing is beneficial to reset circadian homeostasis and attain maximal cancer suppression. Further studies are warranted to investigate the mechanisms underlying the TRF-mediated anti-tumor effect and to explore the therapeutic efficacy of combination treatment of TRF and TIM inhibition or plus chemotherapy in patients with cancer.

### Supplementary Information

**Additional file 1:** **Fig. S1. **TRF inhibits proliferation and migration of lung adenocarcinoma cells. **Fig. S2. **TRF effects on organ pathology in A549 xenograft lung tumorigenesis mouse models. **Fig. S3. **TRF effects on rhythm expression of serum measures in A549 xenograft mice. **Fig. S4. **TRF effects on temporal expressions of metabolites involved in energy metabolism. **Fig. S5. **GPI is not involved in TRF-mediated anti-tumor effect in vitro. **Fig. S6. **Clock genes expressions are altered in lung adenocarcinoma and correlate with survival phenotype. **Fig. S7. **TRF regulates the rhythm expression of circadian genes in multiple tumor cell lines. **Fig. S8. **The effects of TRF on clock genes in the lung and liver tissues. **Table S1. **Circadian parameters for serum measures in xenograft lung tumorigenesis mice related to Fig. S3A-G. **Table S2. **Circadian parameters for serum measures in urethane-administrated mice related to Fig. S3H-K.** Table S3. **Statistical analysis of circadian parameters related to Fig. S4. **Table S4.** Statistical analysis of circadian parameters for genes expression related to Fig. 4D-I, K. **Table S5. **Statistical analysis of circadian parameters for genes levels related to Fig. 5. **Table S6. **Statistical analysis of circadian parameters related to Fig. S7. **Table S7. **Statistical analysis of circadian parameters related to Fig. S8. **Table S8. **The sequences of all primers.

**Additional file 2:** Raw data retrieved from TCGA and GEO.

## Data Availability

This research did not generate new unique reagents. Data needed to evaluate the conclusions are present in the paper, the Supplementary information. Further information and requests for resources and reagents will be made available by the corresponding author.

## Abbreviations

ALDOA: Aldolase, fructose-bisphosphate A
CQ: Chloroquine
CRY1: Cryptochrome circadian regulator 1
CRY2: Cryptochrome circadian regulator 2
FBP1: Fructose-bisphosphatase 1
FBS: Fetal bovine serum
FBXL3: F-box and leucine rich repeat protein 3
GLU: Glucose
GPER1: G protein-coupled estrogen receptor 1
GPI: Glucose-6-phosphate isomerase
HDL-C: High-density lipoprotein cholesterol
HK1: Hexokinase 1
IGF-1: Insulin-like growth factor 1
LDL-C: Low-density lipoprotein cholesterol
PFKP: Phosphofructokinase, platelet
PGK1: Phosphoglycerate kinase 1
RA: Rapamycin
TCHO: Total cholesterol
TG: Triglyceride
TIM: Timeless
TRF: Time-restricted feeding

## References

[1] Sung H, Ferlay J, Siegel RL, Laversanne M, Soerjomataram I, Jemal A, Bray F (2021). Global Cancer Statistics 2020: GLOBOCAN estimates of incidence and mortality worldwide for 36 cancers in 185 countries. CA Cancer J Clin.

[2] Allemani C, Matsuda T, Di Carlo V, Harewood R, Matz M, Niksic M, Bonaventure A, Valkov M, Johnson CJ, Esteve J (2018). Global surveillance of trends in cancer survival 2000–14 (CONCORD-3): analysis of individual records for 37 513 025 patients diagnosed with one of 18 cancers from 322 population-based registries in 71 countries. Lancet.

[3] Pariollaud M, Lamia KA (2020). Cancer in the fourth dimension: what is the impact of circadian disruption?. Cancer Discov.

[4] Shafi AA, Knudsen KE (2019). Cancer and the circadian clock. Cancer Res.

[5] Sulli G, Lam MTY, Panda S (2019). Interplay between circadian clock and cancer: new frontiers for cancer treatment. Trends Cancer.

[6] Papagiannakopoulos T, Bauer MR, Davidson SM, Heimann M, Subbaraj L, Bhutkar A, Bartlebaugh J, Vander Heiden MG, Jacks T (2016). Circadian rhythm disruption promotes lung tumorigenesis. Cell Metab.

[7] Pariollaud M, Ibrahim LH, Irizarry E, Mello RM, Chan AB, Altman BJ, Shaw RJ, Bollong MJ, Wiseman RL, Lamia KA (2022). Circadian disruption enhances HSF1 signaling and tumorigenesis in Kras-driven lung cancer. Sci Adv.

[8] Li MD (2022). Clock-modulated checkpoints in time-restricted eating. Trends Mol Med.

[9] Queiroz JDN, Macedo RCO, Tinsley GM, Reischak-Oliveira A (2021). Time-restricted eating and circadian rhythms: the biological clock is ticking. Crit Rev Food Sci Nutr.

[10] Longo VD, Panda S (2016). Fasting, circadian rhythms, and time-restricted feeding in healthy lifespan. Cell Metab.

[11] Alidadi M, Banach M, Guest PC, Bo S, Jamialahmadi T, Sahebkar A (2021). The effect of caloric restriction and fasting on cancer. Semin Cancer Biol.

[12] Mittelman SD (2020). The role of diet in cancer prevention and chemotherapy efficacy. Annu Rev Nutr.

[13] Acosta-Rodriguez V, Rijo-Ferreira F, Izumo M, Xu P, Wight-Carter M, Green CB, Takahashi JS (2022). Circadian alignment of early onset caloric restriction promotes longevity in male C57BL/6J mice. Science.

[14] Acosta-Rodriguez VA, de Groot MHM, Rijo-Ferreira F, Green CB, Takahashi JS (2017). Mice under caloric restriction self-impose a temporal restriction of food intake as revealed by an automated feeder system. Cell Metab.

[15] Das M, Ellies LG, Kumar D, Sauceda C, Oberg A, Gross E, Mandt T, Newton IG, Kaur M, Sears DD (2021). Time-restricted feeding normalizes hyperinsulinemia to inhibit breast cancer in obese postmenopausal mouse models. Nat Commun.

[16] Li XM, Delaunay F, Dulong S, Claustrat B, Zampera S, Fujii Y, Teboul M, Beau J, Levi F (2010). Cancer inhibition through circadian reprogramming of tumor transcriptome with meal timing. Cancer Res.

[17] Turbitt WJ, Orlandella RM, Gibson JT, Peterson CM, Norian LA (2020). Therapeutic time-restricted feeding reduces renal tumor bioluminescence in mice but fails to improve anti-CTLA-4 efficacy. Anticancer Res.

[18] Wu MW, Li XM, Xian LJ, Levi F (2004). Effects of meal timing on tumor progression in mice. Life Sci.

[19] Yan L, Sundaram S, Mehus AA, Picklo MJ (2019). Time-restricted feeding attenuates high-fat diet-enhanced spontaneous metastasis of Lewis lung carcinoma in mice. Anticancer Res.

[20] Das M, Webster NJG (2022). Obesity, cancer risk, and time-restricted eating. Cancer Metastasis Rev.

[21] Filipski E, Innominato PF, Wu M, Li XM, Iacobelli S, Xian LJ, Levi F (2005). Effects of light and food schedules on liver and tumor molecular clocks in mice. J Natl Cancer Inst.

[22] Leman AR, Noguchi E (2012). Local and global functions of Timeless and Tipin in replication fork protection. Cell Cycle.

[23] McFarlane RJ, Mian S, Dalgaard JZ (2010). The many facets of the Tim-Tipin protein families’ roles in chromosome biology. Cell Cycle.

[24] Yin H, Wang Z, Wang D, Nuer M, Han M, Ren P, Ma S, Lin C, Chen J, Xian H (2023). TIMELESS promotes the proliferation and migration of lung adenocarcinoma cells by activating EGFR through AMPK and SPHK1 regulation. Eur J Pharmacol..

[25] Yoshida K, Sato M, Hase T, Elshazley M, Yamashita R, Usami N, Taniguchi T, Yokoi K, Nakamura S, Kondo M (2013). TIMELESS is overexpressed in lung cancer and its expression correlates with poor patient survival. Cancer Sci.

[26] Shi D, Wu J, Wu Y, Lin X, Xu C, Lian X (2021). High-fat diet-related obesity promotes urethane-induced lung tumorigenesis in C57BL/6J mice. Front Oncol.

[27] Shi D, Han T, Chu X, Lu H, Yang X, Zi T, Zhao Y, Wang X, Liu Z, Ruan J (2021). An isocaloric moderately high-fat diet extends lifespan in male rats and Drosophila. Cell Metab..

[28] Xin H, Deng F, Zhou M, Huang R, Ma X, Tian H, Tan Y, Chen X, Deng D, Shui G (2021). A multi-tissue multi-omics analysis reveals distinct kineztics in entrainment of diurnal transcriptomes by inverted feeding. iScience.

[29] Vander Heiden MG, Cantley LC, Thompson CB (2009). Understanding the Warburg effect: the metabolic requirements of cell proliferation. Science.

[30] Gridelli C, Rossi A, Carbone DP, Guarize J, Karachaliou N, Mok T, Petrella F, Spaggiari L, Rosell R (2015). Non-small-cell lung cancer. Nat Rev Dis Primers.

[31] Yin Z, Klionsky DJ (2022). Intermittent time-restricted feeding promotes longevity through circadian autophagy. Autophagy.

[32] Jamshed H, Beyl RA, Della Manna DL, Yang ES, Ravussin E, Peterson CM (2019). Early time-restricted feeding improves 24-hour glucose levels and affects markers of the circadian clock, aging, and autophagy in humans. Nutrients.

[33] Li X, He S, Ma B (2020). Autophagy and autophagy-related proteins in cancer. Mol Cancer.

[34] Poillet-Perez L, White E (2019). Role of tumor and host autophagy in cancer metabolism. Genes Dev.

[35] Masri S, Sassone-Corsi P (2018). The emerging link between cancer, metabolism, and circadian rhythms. Nat Med.

[36] Kinouchi K, Sassone-Corsi P (2020). Metabolic rivalry: circadian homeostasis and tumorigenesis. Nat Rev Cancer.

[37] Almoosawi S, Vingeliene S, Gachon F, Voortman T, Palla L, Johnston JD, Van Dam RM, Darimont C, Karagounis LG (2019). Chronotype: implications for epidemiologic studies on chrono-nutrition and cardiometabolic health. Adv Nutr.

[38] Asher G, Sassone-Corsi P (2015). Time for food: the intimate interplay between nutrition, metabolism, and the circadian clock. Cell.

[39] Hawley JA, Sassone-Corsi P, Zierath JR (2020). Chrono-nutrition for the prevention and treatment of obesity and type 2 diabetes: from mice to men. Diabetologia.

[40] Adafer R, Messaadi W, Meddahi M, Patey A, Haderbache A, Bayen S, Messaadi N (2020). Food timing, circadian rhythm and chrononutrition: a systematic review of time-restricted eating’s effects on human health. Nutrients.

[41] Hoddy KK, Marlatt KL, Cetinkaya H, Ravussin E (2020). Intermittent fasting and metabolic health: from religious fast to time-restricted feeding. Obesity (Silver Spring).

[42] Lewis P, Oster H, Korf HW, Foster RG, Erren TC (2020). Food as a circadian time cue - evidence from human studies. Nat Rev Endocrinol.

[43] Yan L, Rust BM, Picklo MJ (2020). Plasma metabolomic changes in mice with time-restricted feeding-attenuated spontaneous metastasis of Lewis lung carcinoma. Anticancer Res.

[44] Hatori M, Vollmers C, Zarrinpar A, DiTacchio L, Bushong EA, Gill S, Leblanc M, Chaix A, Joens M, Fitzpatrick JA (2012). Time-restricted feeding without reducing caloric intake prevents metabolic diseases in mice fed a high-fat diet. Cell Metab.

[45] Lundell LS, Parr EB, Devlin BL, Ingerslev LR, Altintas A, Sato S, Sassone-Corsi P, Barres R, Zierath JR, Hawley JA (2020). Time-restricted feeding alters lipid and amino acid metabolite rhythmicity without perturbing clock gene expression. Nat Commun.

[46] Cong J, Wang X, Zheng X, Wang D, Fu B, Sun R, Tian Z, Wei H (2018). Dysfunction of natural killer cells by FBP1-induced inhibition of glycolysis during lung cancer progression. Cell Metab.

[47] Kathagen-Buhmann A, Maire CL, Weller J, Schulte A, Matschke J, Holz M, Ligon KL, Glatzel M, Westphal M, Lamszus K (2018). The secreted glycolytic enzyme GPI/AMF stimulates glioblastoma cell migration and invasion in an autocrine fashion but can have anti-proliferative effects. Neuro Oncol.

[48] Vander Heiden MG, DeBerardinis RJ (2017). Understanding the intersections between metabolism and cancer biology. Cell.

[49] Dobashi Y, Watanabe H, Sato Y, Hirashima S, Yanagawa T, Matsubara H, Ooi A (2006). Differential expression and pathological significance of autocrine motility factor/glucose-6-phosphate isomerase expression in human lung carcinomas. J Pathol.

[50] Santos HO, Genario R, Tinsley GM, Ribeiro P, Carteri RB, Coelho-Ravagnani CF, Mota JF (2022). A scoping review of intermittent fasting, chronobiology, and metabolism. Am J Clin Nutr.

[51] Ulgherait M, Midoun AM, Park SJ, Gatto JA, Tener SJ, Siewert J, Klickstein N, Canman JC, Ja WW, Shirasu-Hiza M (2021). Circadian autophagy drives iTRF-mediated longevity. Nature.

[52] Zhang W, He W, Shi Y, Zhao J, Liu S, Zhang F, Yang J, Xie C, Zhang Y (2017). Aberrant TIMELESS expression is associated with poor clinical survival and lymph node metastasis in early-stage cervical carcinoma. Int J Oncol.

[53] Bianco JN, Bergoglio V, Lin YL, Pillaire MJ, Schmitz AL, Gilhodes J, Lusque A, Mazieres J, Lacroix-Triki M, Roumeliotis TI (2019). Overexpression of Claspin and Timeless protects cancer cells from replication stress in a checkpoint-independent manner. Nat Commun.

[54] Zhou J, Zhang Y, Zou X, Kuai L, Wang L, Wang J, Shen F, Hu J, Zhang X, Huang Y (2020). Aberrantly expressed Timeless regulates cell proliferation and cisplatin efficacy in cervical cancer. Hum Gene Ther.

[55] Li B, Mu L, Li Y, Xia K, Yang Y, Aman S, Ahmad B, Li S, Wu H (2021). TIMELESS inhibits breast cancer cell invasion and metastasis by down-regulating the expression of MMP9. Cancer Cell Int.

[56] Colangelo T, Carbone A, Mazzarelli F, Cuttano R, Dama E, Nittoli T, Albanesi J, Barisciano G, Forte N, Palumbo O (2022). Loss of circadian gene Timeless induces EMT and tumor progression in colorectal cancer via Zeb1-dependent mechanism. Cell Death Differ.

[57] Damato AR, Herzog ED (2022). Circadian clock synchrony and chronotherapy opportunities in cancer treatment. Semin Cell Dev Biol.

[58] Li J, Chen Q, Shi D, Lian X (2022). Combined time-restricted feeding and cisplatin enhance the anti-tumor effects in cisplatin-resistant and -sensitive lung cancer cells. Med Oncol.

[59] Levy JMM, Towers CG, Thorburn A (2017). Targeting autophagy in cancer. Nat Rev Cancer.

[60] Pietrocola F, Pol J, Vacchelli E, Rao S, Enot DP, Baracco EE, Levesque S, Castoldi F, Jacquelot N, Yamazaki T (2016). Caloric restriction mimetics enhance anticancer immunosurveillance. Cancer Cell.

[61] Sadeghian M, Rahmani S, Khalesi S, Hejazi E (2021). A review of fasting effects on the response of cancer to chemotherapy. Clin Nutr.

