# Supplementary material for: Six-hour time-restricted feeding inhibits lung cancer progression and reshapes circadian metabolism
Source: BMC Med. 2023 Nov 3;21:417. doi: 10.1186/s12916-023-03131-y (PMC10625271; doi:10.1186/s12916-023-03131-y)

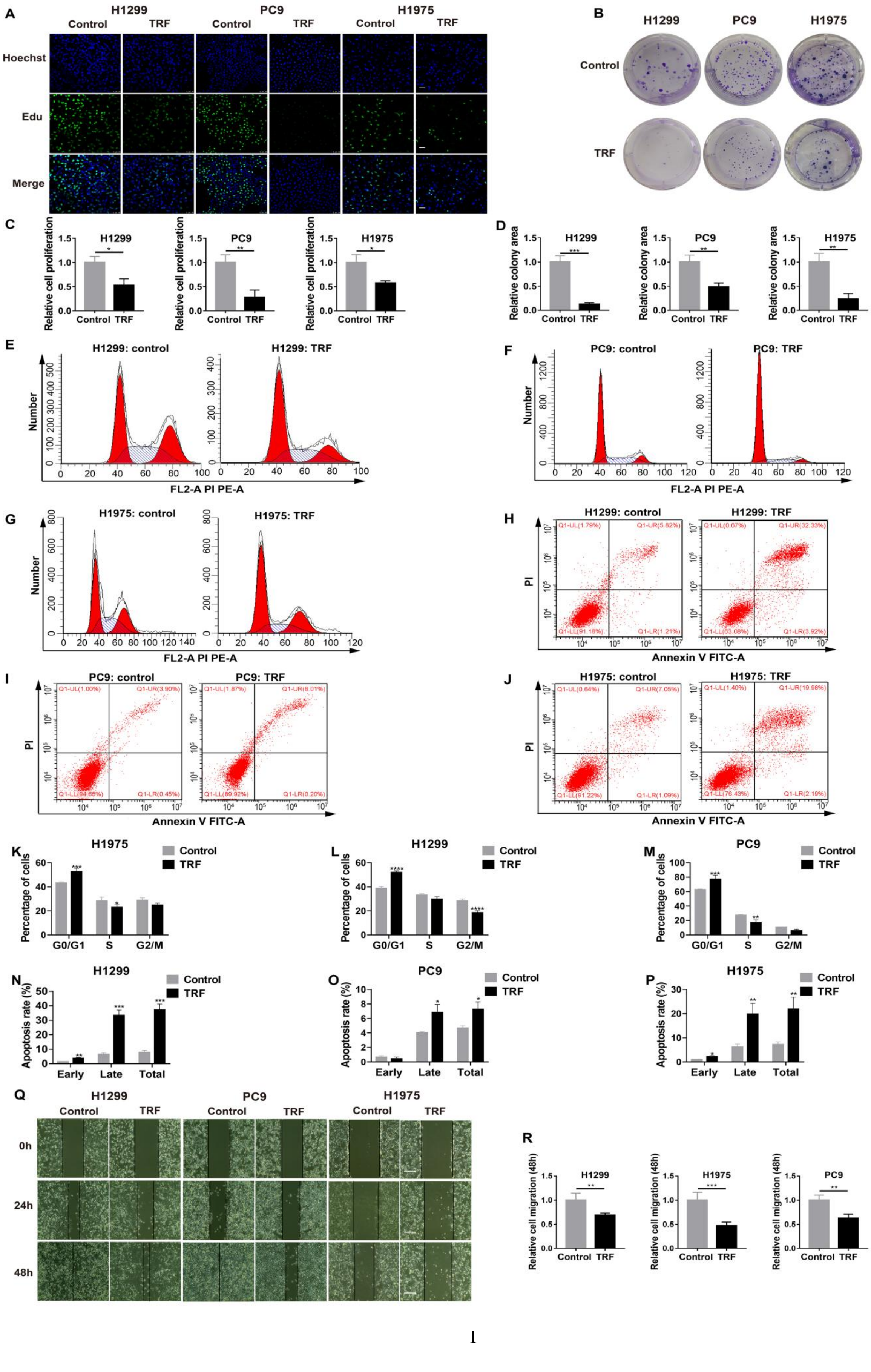
A
H1299
PC9
H1975
Control
TRF
Hoechst
Edu
Merge
B
Control
TRF
C
Relative cell proliferation
D
Relative colony area
E
H1299: control
H1299: TRF
Number
FL2-A PI PE-A
F
PC9: control
PC9: TRF
G
H1975: control
H1975: TRF
H
Annexin V FITC-A
PI
I
PC9: control
PC9: TRF
J
H1975: control
H1975: TRF
K
L
M
Percentage of cells
G0/G1
S
G2/M
N
O
P
Apoptosis rate (%)
Early
Late
Total
Q
0h
24h
48h
R
Relative cell migration (48h)

Fig. S1. TRF inhibits proliferation and migration of lung adenocarcinoma cells.

(A) Representative images of Edu assays in the H1299, PC9 and H1975 cells in the control group and the TRF group (scale bar = 50 μm). **(B)** Representative images of colony formation assays in the H1299, PC9 and H1975 cells exposed to the TRF medium. **(C)** Quantification of the Edu analysis in the H1299, PC9 and H1975 cells (n = 3). **(D)** Quantification of the colony formation assays in the H1299, PC9 and H1975 cells (n = 3). **(E-G)** Representative images of flow cytometry assay to detect the cell cycle in the **(E)** H1299, **(F)** PC9 and **(G)** H1975 cells treated with TRF intervention. **(H-J)** Representative images of flow cytometry assay to determine the cell apoptosis in the **(H)** H1299, **(I)** PC9 and **(J)** H1975 cells treated with TRF intervention. **(K-M)** Quantification of the cell cycle in the **(K)** H1299, **(L)** PC9 and **(M)** H1975 cells (n = 3). **(N-P)** Quantification results generated from cell apoptosis in the **(N)** H1299, **(O)** PC9 and **(P)** H1975 cells (n = 3). **(Q)** Representative images of wound healing experiment in the H1299, PC9 and H1975 cells subjected with a control or a TRF paradigm (scale bar = 100 μm). **(R)** Quantitative analysis of wound healing detection in the H1299, PC9 and H1975 cells (n = 4). Data were analyzed by two-tailed Student's t test or two-way ANOVA with Tukey's post hoc test. Error bars, when present, show the SD. *P < 0.05; **P < 0.01; ***P < 0.001; ****P < 0.0001

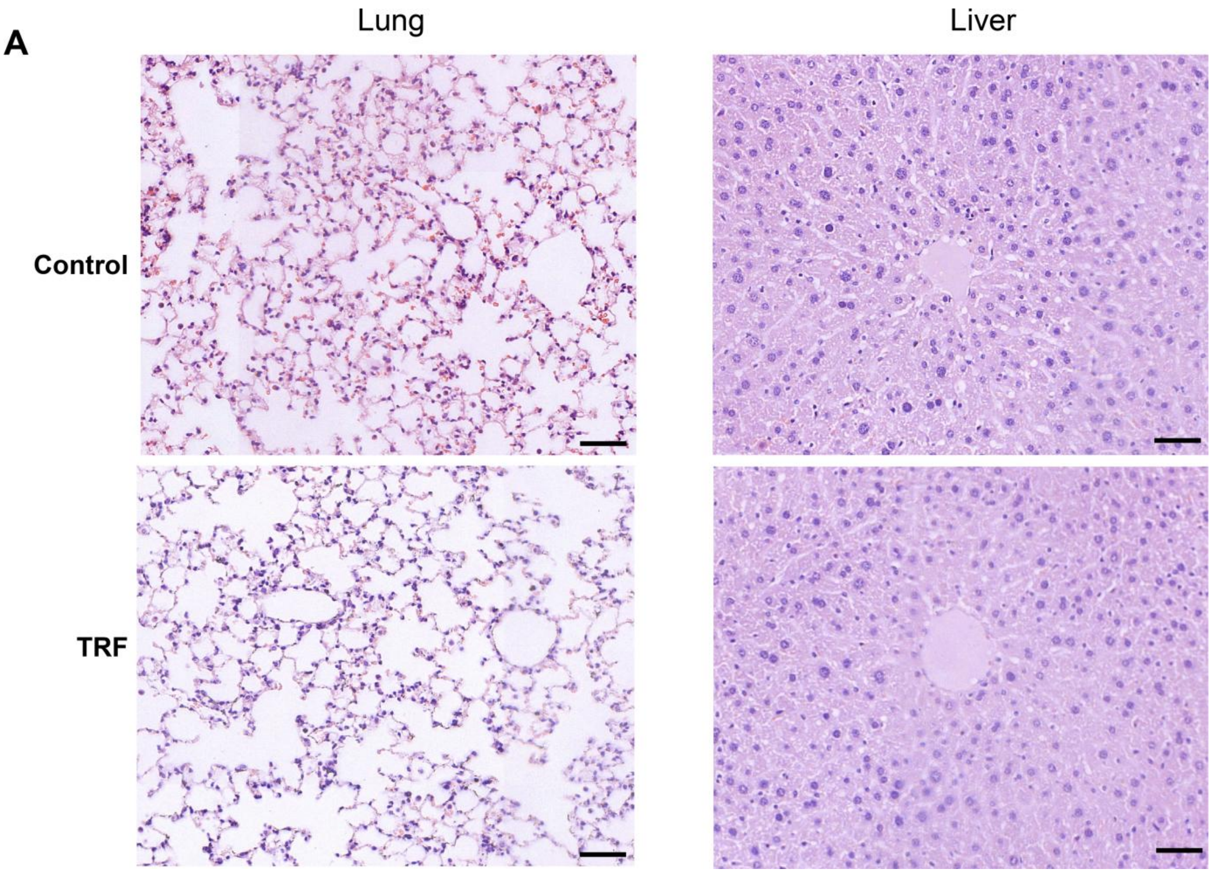

Fig. S2. TRF effects on organ pathology in A549 xenograft lung tumorigenesis mouse models.

(A) Histopathology. Representative H&E staining of lung and liver tissues in A549 xenograft model upon control and TRF (scale bar = 50 μm).

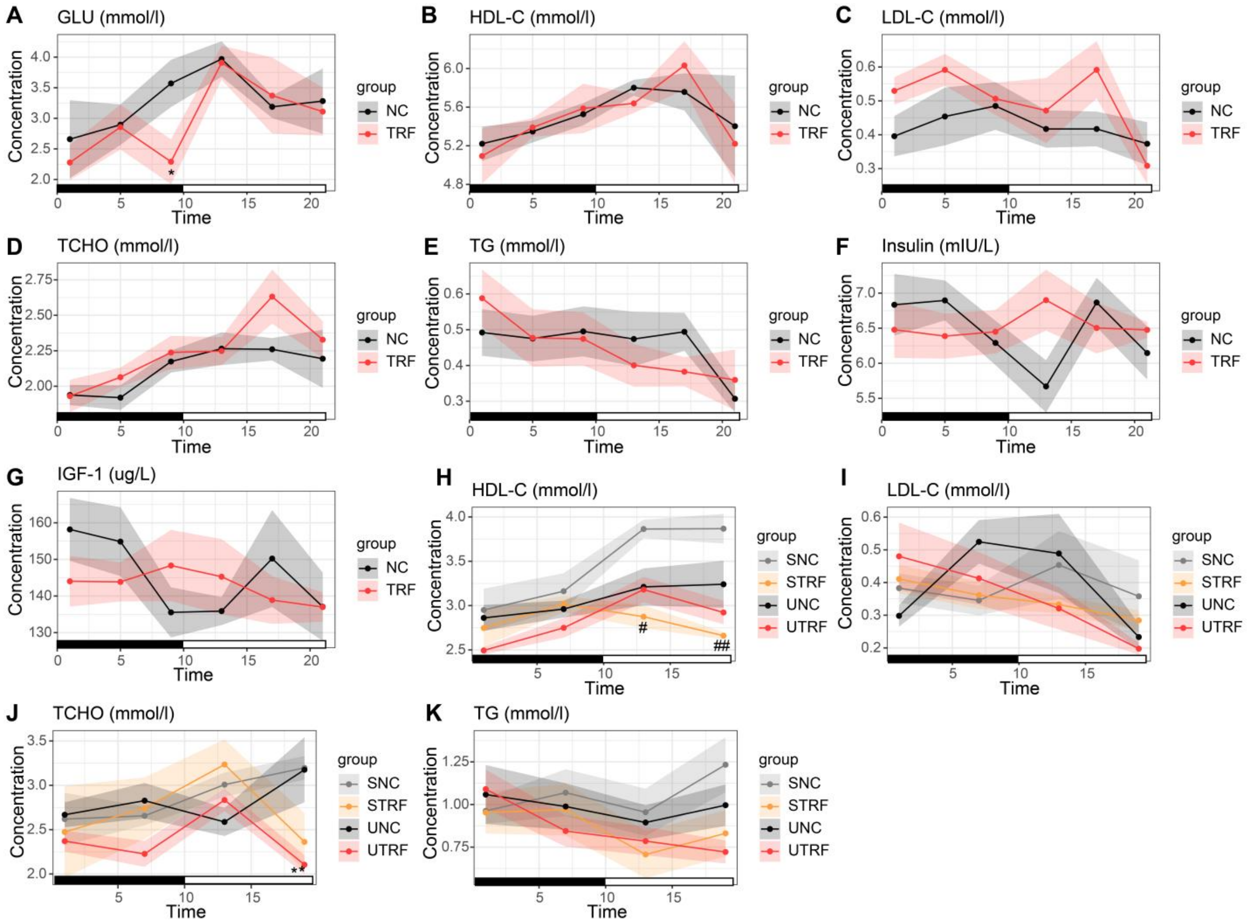

Fig. S3. TRF effects on rhythm expression of serum measures in A549 xenograft mice.

(A–G) Serum chemistry measures over 24h in A549 xenograft model. Serum concentrations of **(A)** glucose (GLU), **(B)** high-density lipoprotein cholesterol (HDL-C), **(C)** low-density lipoprotein cholesterol (LDL-C), **(D)** total cholesterol (TCHO), **(E)** triglycerides (TG), **(F)** Insulin, and **(G)** insulin like growth factor 1 (IGF-1) (NC, n = 5–6 mice each time; TRF, n = 5-6 mice each time). Shaded area showed standard error of mean (SEM). Black axis indicated period of lights off. **(H–K)** Serum parameters over 24h in urethane-administered mice model. Serum concentrations of **(H)** HDL-C, **(I)** LDL-C, **(J)** TCHO, **(K)** TG (SNC, n = 5 mice each time; STRF, n = 3-4 mice each time; UNC, n = 6-7 mice each time; UTRF, n = 5-6 mice each time). Saline-treated normal control, SNC; saline-treated time-restricted feeding, STRF; urethane-treated normal control, UNC; urethane-treated time-restricted feeding, UTRF. Shaded area represented standard error of mean (SEM). Black axis indicated period of lights off. Data were

analyzed by MetaCycle or two-way ANOVA with Tukey post-hoc test. See also Data S1. *Compared with the UNC group, *$P < 0.05$; **$P < 0.01$; ***$P < 0.001$; ****$P < 0.0001$; #Compared with the SNC group, #$P < 0.05$; ##$P < 0.01$; ###$P < 0.001$; ####$P < 0.0001$.

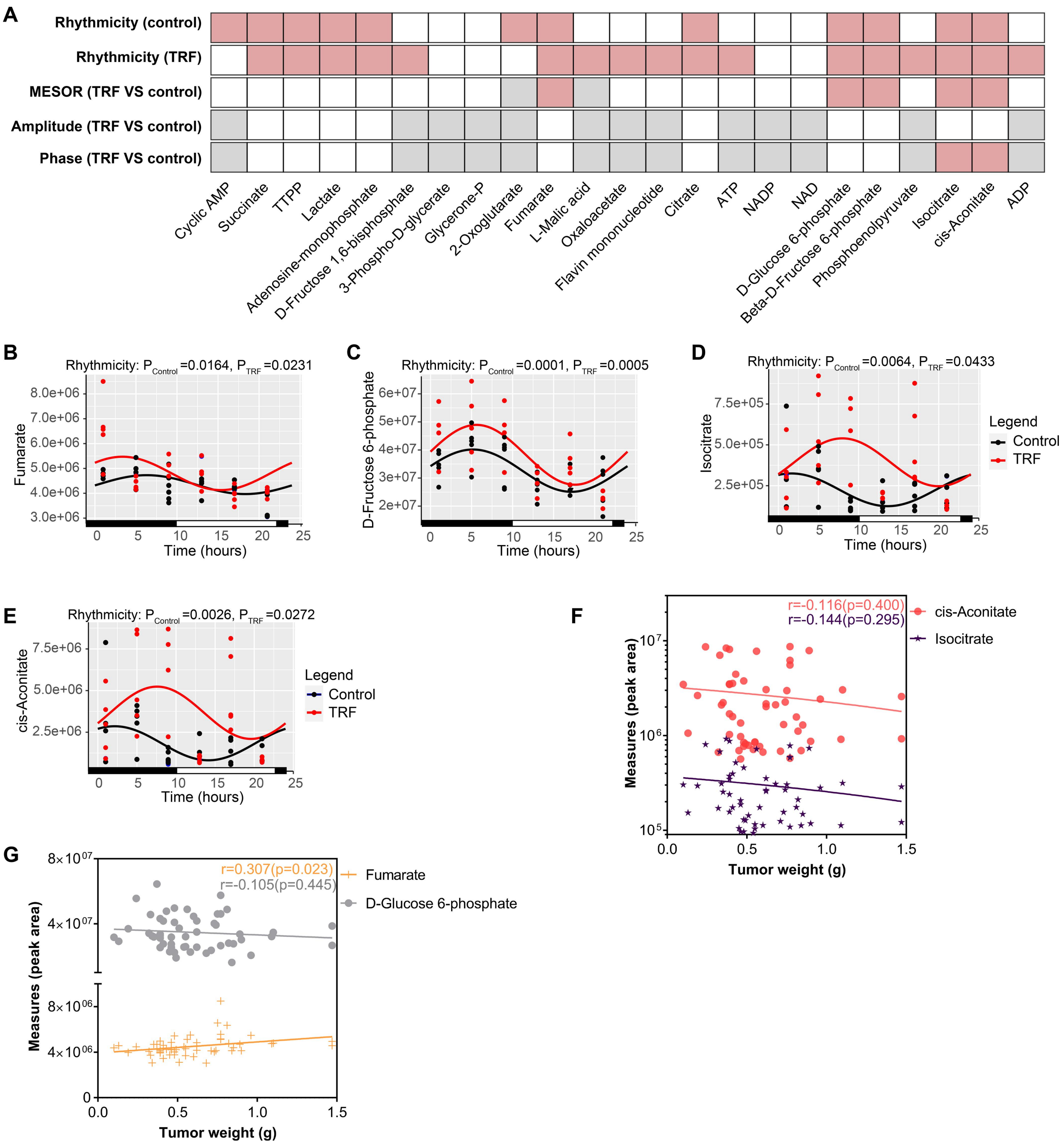

Fig. S4. TRF effects on temporal expressions of metabolites involved in energy metabolism.

(A) Rhythm results of energy metabolites analyzed by circacompare. Circadian metabolites were color-coded. Mesor, amplitude and phase in the TRF group versus control were color-coded, with red color showing differences, with light grey showing metabolites that could not be compared, and white showing non-significant metabolites. **(B-E)** Abundance of metabolites in tumor tissues from mouse xenograft model collected

at different times (ZT1, 5, 9, 13, 17, 21) over 24h period, including **(B)** fumarate, **(C)** d-fructose 6-bisphosphate, **(D)** isocitrate and **(E)** cis-aconitate. (Control: n = 5-6 mice each time; TRF: n = 4-5 mice each time). ZT0 indicated 10 pm which was the start of the feeding of TRF intervention during experiment. **(F)** Correlation analysis among metabolites of cis-aconitate, isocitrate and tumor weight (n = 55 mice). **(G)** Correlation analysis among metabolites of fumarate, d-frucose 6-phosphate and tumor weight (n = 55 mice). Data were analyzed and visualized by the circacompare or analyzed by Pearson correlation analysis. Error bars, when present, show the SEM.

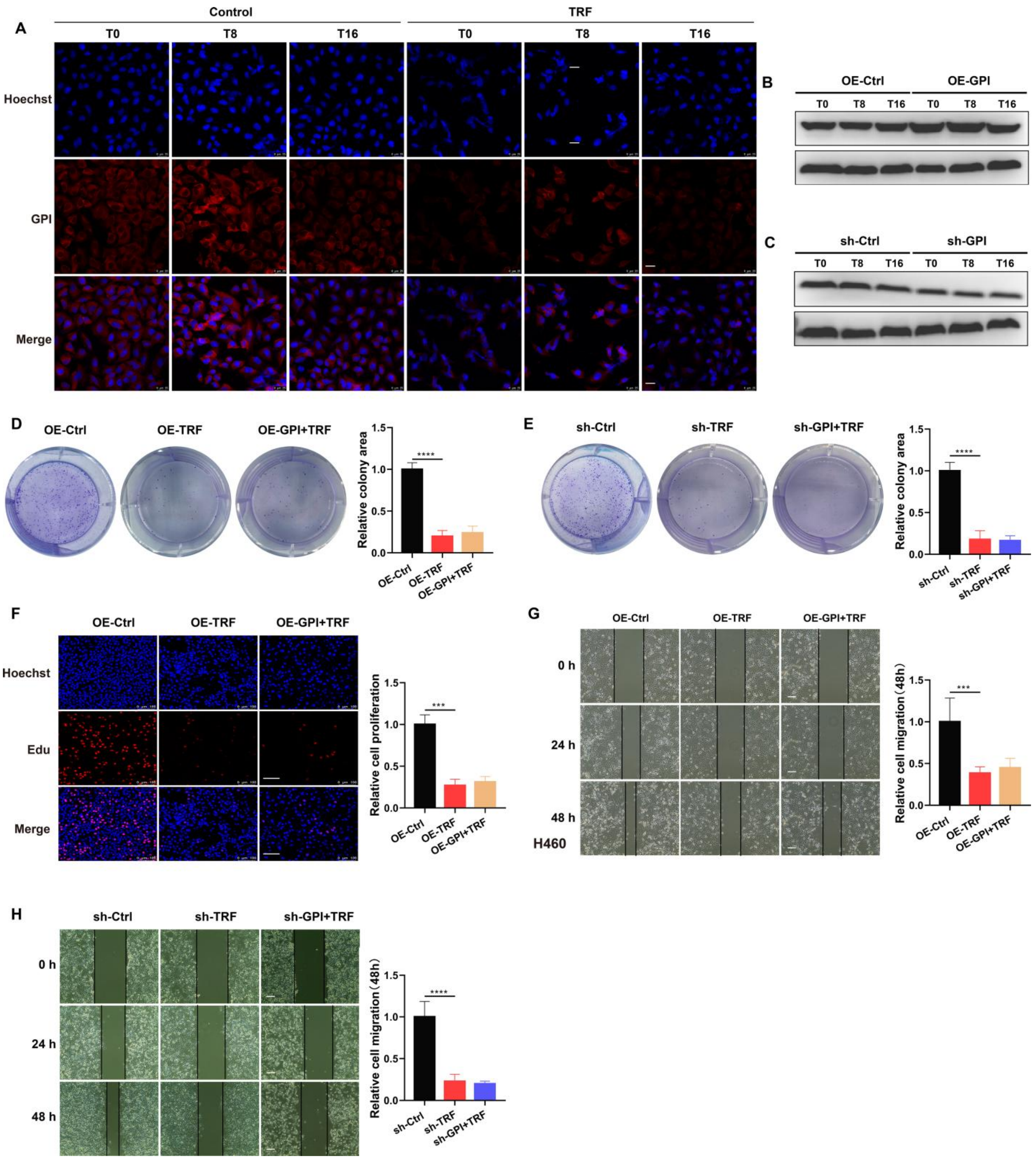

Fig. S5. GPI is not involved in TRF-mediated anti-tumor effect in vitro.

(A) Protein expression of GPI with TRF or not in A549 cell was examined by immunohistochemical staining. (Scale bar = 25 μm). **(B-C)** Protein expression of GPI was detected by western blot in A549 stable cell lines. Representative blots are shown for GPI protein in **(B)** GPI overexpression stable cell, and **(C)** GPI knockdown stable cell

(n = 3). **(D-E)** Colony formation assay. Colony formation assay to determine cell proliferation upon TRF in **(D)** GPI overexpression stable cell, and **(E)** GPI knockdown stable cell. Left: representative images; Right: quantification data (n = 3). **(F)** Edu assay. Edu assay to examine cell proliferation upon TRF in GPI overexpression stable cell. Left: representative images; Right: quantification data (n = 3). (Scale bar = 100 μm). **(G-H)** Wound healing assay. Wound healing assay to determine cell migration ability upon TRF in **(G)** GPI overexpression stable cell, and **(H)** GPI knockdown stable cell. Left: representative images; Right: quantification data (n = 5). (Scale bar = 50 μm). Data were analyzed by two-way ANOVA with Tukey post-hoc test. Error bars, when present, show the SD. *$P < 0.05$; **$P < 0.01$; ***$P < 0.001$; ****$P < 0.0001$.

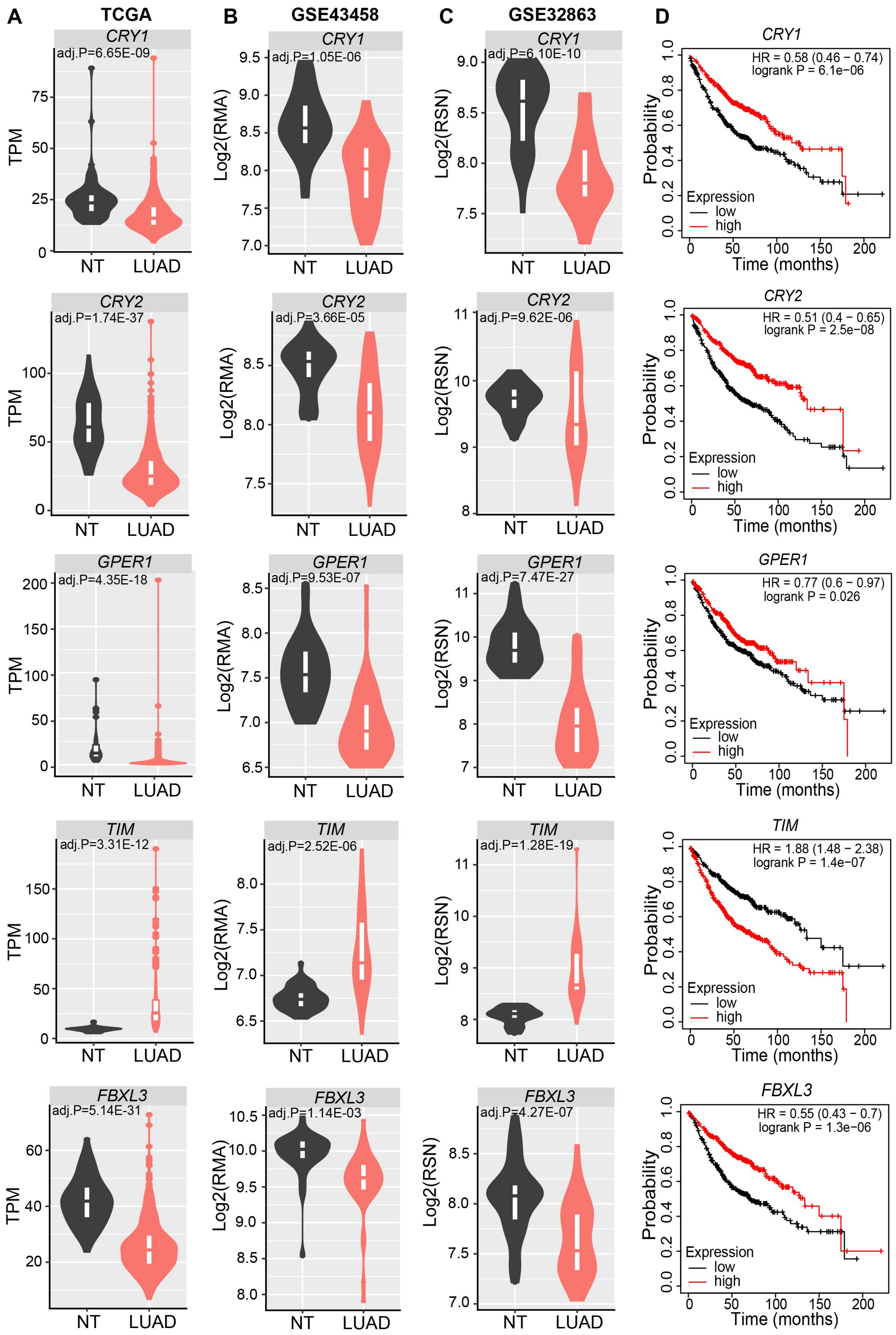
A
TCGA
B
GSE43458
C
GSE32863
D
CRY1
adj.P=6.65E-09
adj.P=1.05E-06
adj.P=6.10E-10
HR = 0.58 (0.46 − 0.74)
logrank P = 6.1e−06
CRY2
adj.P=1.74E-37
adj.P=3.66E-05
adj.P=9.62E-06
HR = 0.51 (0.4 − 0.65)
logrank P = 2.5e−08
GPER1
adj.P=4.35E-18
adj.P=9.53E-07
adj.P=7.47E-27
HR = 0.77 (0.6 − 0.97)
logrank P = 0.026
TIM
adj.P=3.31E-12
adj.P=2.52E-06
adj.P=1.28E-19
HR = 1.88 (1.48 − 2.38)
logrank P = 1.4e−07
FBXL3
adj.P=5.14E-31
adj.P=1.14E-03
adj.P=4.27E-07
HR = 0.55 (0.43 − 0.7)
logrank P = 1.3e−06
TPM
Log2(RMA)
Log2(RSN)
Probability
NT
LUAD
Expression
low
high
Time (months)

Fig. S6. Clock genes expressions are altered in lung adenocarcinoma and correlate with survival phenotype.

(A) The mRNA expression levels of clock genes in lung adenocarcinoma and normal tissues were derived from TCGA dataset. Expression levels are showed with TPM (NT: n = 59; LUAD: n = 513). **(B)** The mRNA expression levels in non-smokers of lung adenocarcinoma and normal tissues were obtained from GEO dataset of GSE43458. Expression levels are showed with log2(RMA) (NT: n = 30; LUAD: n = 40). **(C)** The mRNA expression levels in non-smokers of lung adenocarcinoma and normal tissues were derived from GEO dataset of GSE32863. Expression levels are showed with log2(RSN) (NT: n = 30; LUAD: n = 29). **(D)** Kaplan–Meier curves for overall survival were plotted by Kaplan-Meier Plotter (www.kmplot.com) in lung adenocarcinoma. CRY1 (209674_at; low, n = 360; high, n = 359), CRY2 (212695_at; low, n = 360; high, n=359), GPER1 (210640_s_at; low, n=361; high, n=358), TIMELESS (203046_s_at, low, n = 360; high, n=359), FBXL3 (225132_at; low, n = 337; high, n = 335). Hazard ratio (HR) with 95% confidence interval and a log-rank P value for survival analysis was computed using univariate cox regression. TCGA were analyzed with limma and GEO datasets were analyzed with GEO2R.

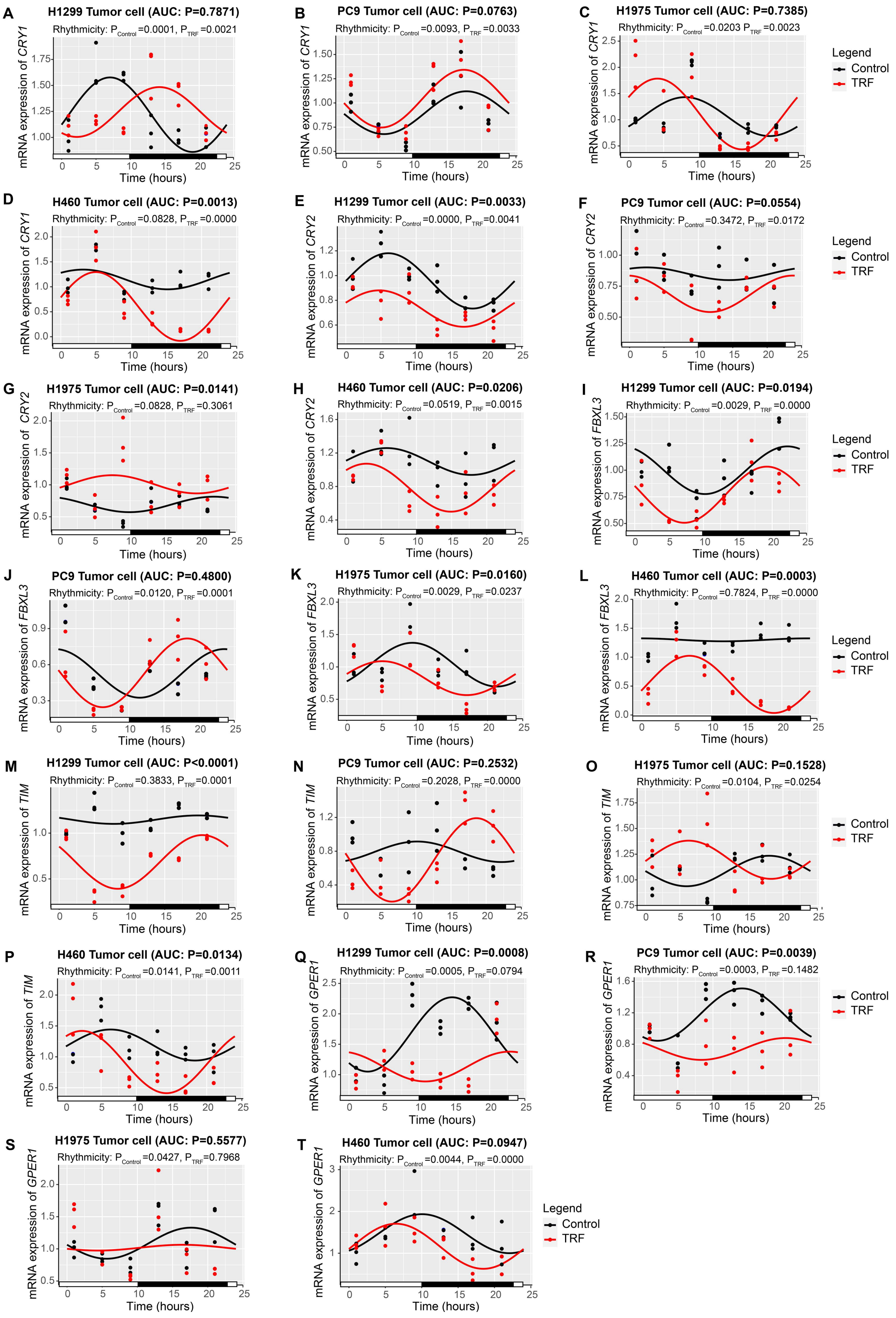
A
H1299 Tumor cell (AUC: P=0.7871)
Rhythmicity: PControl =0.0001, PTRF =0.0021
mRNA expression of CRY1
Time (hours)
B
PC9 Tumor cell (AUC: P=0.0763)
Rhythmicity: PControl =0.0093, PTRF =0.0033
mRNA expression of CRY1
Time (hours)
C
H1975 Tumor cell (AUC: P=0.7385)
Rhythmicity: PControl =0.0203 PTRF =0.0023
mRNA expression of CRY1
Time (hours)
Legend
Control
TRF
D
H460 Tumor cell (AUC: P=0.0013)
Rhythmicity: PControl =0.0828, PTRF =0.0000
mRNA expression of CRY1
Time (hours)
E
H1299 Tumor cell (AUC: P=0.0033)
Rhythmicity: PControl =0.0000, PTRF =0.0041
mRNA expression of CRY2
Time (hours)
F
PC9 Tumor cell (AUC: P=0.0554)
Rhythmicity: PControl =0.3472, PTRF =0.0172
mRNA expression of CRY2
Time (hours)
Legend
Control
TRF
G
H1975 Tumor cell (AUC: P=0.0141)
Rhythmicity: PControl =0.0828, PTRF =0.3061
mRNA expression of CRY2
Time (hours)
H
H460 Tumor cell (AUC: P=0.0206)
Rhythmicity: PControl =0.0519, PTRF =0.0015
mRNA expression of CRY2
Time (hours)
I
H1299 Tumor cell (AUC: P=0.0194)
Rhythmicity: PControl =0.0029, PTRF =0.0000
mRNA expression of FBXL3
Time (hours)
Legend
Control
TRF
J
PC9 Tumor cell (AUC: P=0.4800)
Rhythmicity: PControl =0.0120, PTRF =0.0001
mRNA expression of FBXL3
Time (hours)
K
H1975 Tumor cell (AUC: P=0.0160)
Rhythmicity: PControl =0.0029, PTRF =0.0237
mRNA expression of FBXL3
Time (hours)
L
H460 Tumor cell (AUC: P=0.0003)
Rhythmicity: PControl =0.7824, PTRF =0.0000
mRNA expression of FBXL3
Time (hours)
Legend
Control
TRF
M
H1299 Tumor cell (AUC: P<0.0001)
Rhythmicity: PControl =0.3833, PTRF =0.0001
mRNA expression of TIM
Time (hours)
N
PC9 Tumor cell (AUC: P=0.2532)
Rhythmicity: PControl =0.2028, PTRF =0.0000
mRNA expression of TIM
Time (hours)
O
H1975 Tumor cell (AUC: P=0.1528)
Rhythmicity: PControl =0.0104, PTRF =0.0254
mRNA expression of TIM
Time (hours)
Control
TRF
P
H460 Tumor cell (AUC: P=0.0134)
Rhythmicity: PControl =0.0141, PTRF =0.0011
mRNA expression of TIM
Time (hours)
Q
H1299 Tumor cell (AUC: P=0.0008)
Rhythmicity: PControl =0.0005, PTRF =0.0794
mRNA expression of GPER1
Time (hours)
R
PC9 Tumor cell (AUC: P=0.0039)
Rhythmicity: PControl =0.0003, PTRF =0.1482
mRNA expression of GPER1
Time (hours)
Control
TRF
S
H1975 Tumor cell (AUC: P=0.5577)
Rhythmicity: PControl =0.0427, PTRF =0.7968
mRNA expression of GPER1
Time (hours)
T
H460 Tumor cell (AUC: P=0.0947)
Rhythmicity: PControl =0.0044, PTRF =0.0000
mRNA expression of GPER1
Time (hours)
Legend
Control
TRF

Fig. S7. TRF regulates the rhythm expression of circadian genes in multiple tumor cell lines.

(A-T) mRNA expressions of Clock genes detected by RT-PCR in distinct lung cancer cell lines at different times (ZT1, 5, 9, 13, 17, 21) over 24h period. ZT0 indicated the end of the two cycles of 24h TRF intervention in vitro. **(A-D)** mRNA expressions of *CRY1* genes in the **(A)** H1299, **(B)** PC9, **(C)** H1975, **(D)** H460 tumor cells. **(E-H)** mRNA expressions of *CRY2* genes in the **(E)** H1299, **(F)** PC9, **(G)** H1975, **(H)** H460 tumor cells. **(I-L)** mRNA expressions of *FBXL3* genes in the **(I)** H1299, **(J)** PC9, **(K)** H1975, **(L)** H460 tumor cells. **(M-P)** mRNA expressions of *TIM* genes in the **(M)** H1299, **(N)** PC9, **(O)** H1975, **(P)** H460 tumor cells. **(Q-T)** mRNA expressions of *GPER1* genes in the **(Q)** H1299, **(R)** PC9, **(S)** H1975, **(T)** H460 tumor cells. (Control: n = 3 each time; TRF: n = 3 each time). Data were visualized by the circacompare. Data were analyzed by circacompare or AUC. Error bars, when present, show the SEM.

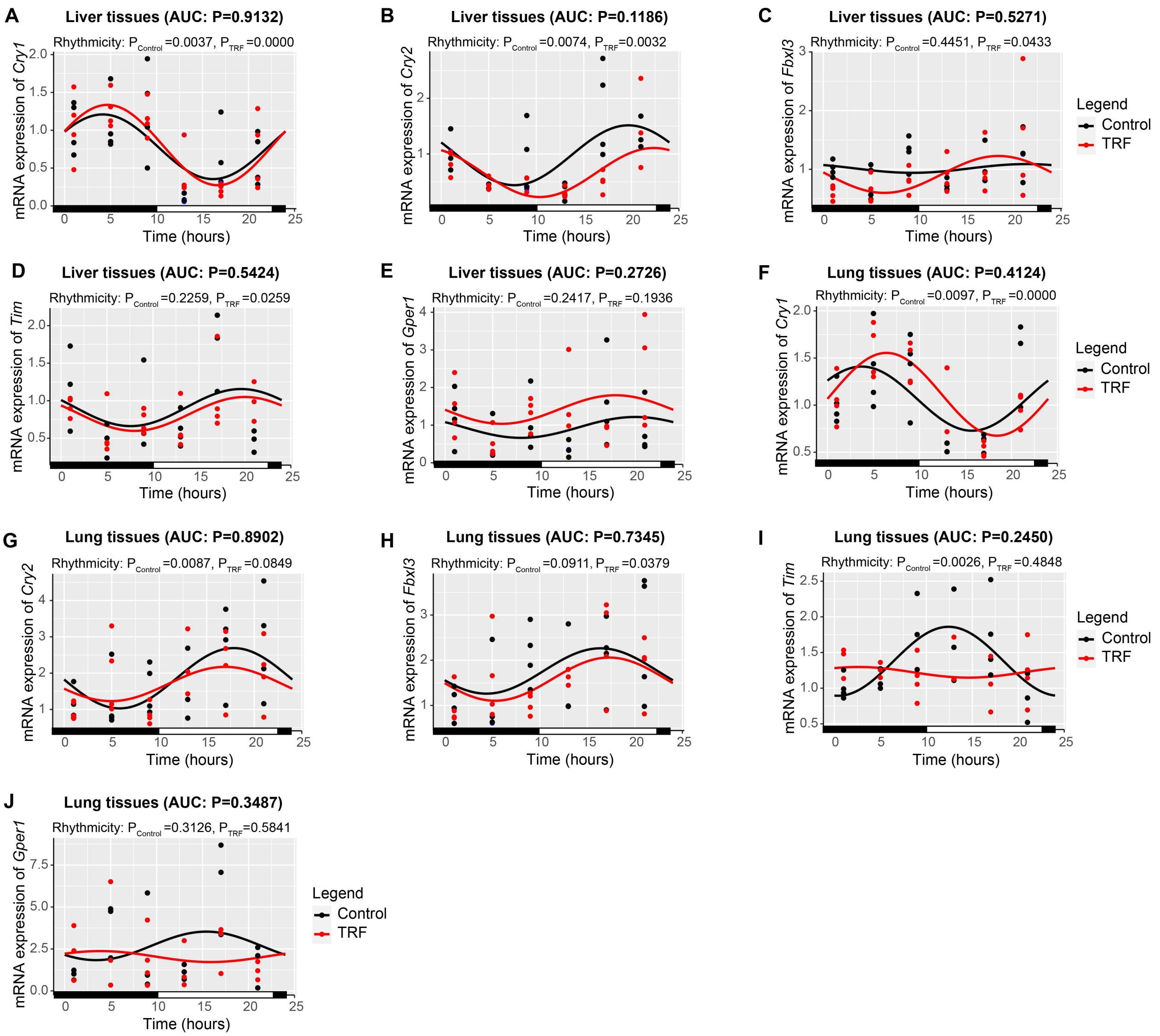

Fig. S8. The effects of TRF on clock genes in the lung and liver tissues.

(A-J) mRNA expressions of clock genes in distinct tissues prepared from A549 xenograft-bearing mouse at different times (ZT1, 5, 9, 13, 17, 21) over 24h. ZT0 indicated 10 pm which was the start of the feeding of TRF intervention. **(A-E)** mRNA expressions of genes in liver tissues, including **(A)** *Cry1,* **(B)** *Cry2*, **(C)** *Fbxl3*, **(D)** *Tim* and **(E)** *Gper1*. **(F-J)** Genes expressions in lung tissues, including **(F)** *Cry1,* **(G)** *Cry2*, **(H)** *Fbxl3*, **(I)** *Tim* and **(J)** *Gper1.* (Control: n = 3-4 each time; TRF: n= 3-4 each time). Data were visualized by the circacompare. Data were analyzed by circacompare or AUC. Error bars, when present, show the SEM.

Table S1. Circadian parameters for serum measures in xenograft lung tumorigenesis mice related to Fig. S3A-G.

Statistical analysis for Fig. S3A-G									
Measures	Group	Period	Rhythmicity (pVal)	Mesor	Mesor (pVal)[a]	Amplitude	Amplitude (pVal)	Phase	Phase (pVal)
GLU	Control	24	0.0406	3.2607		0.515		12.9678	
	TRF	24	0.0224	2.9778	0.248	0.591	0.8257	15.4433	0.3023
TG	Control	24	0.1155	0.4544		0.0588		9.2124	
	TRF	24	0.0509	0.4459	NA	0.0806	NA	3.9928	NA
TCHO	Control	24	0.0087	2.127		0.1895		15.1756	
	TRF	24	0.0011	2.2465	0.0989	0.2686	0.4333	16.0545	0.6173
HDL-C	Control	24	0.0508	5.5079		0.2962		14.1112	
	TRF	24	0.018	5.5071	NA	0.3647	NA	13.9228	NA
LDL-C	Control	24	0.1906	0.4238		0.0468		8.6086	
	TRF	24	0.2073	0.5055	NA	0.0552	NA	8.1304	NA
Insulin	Control	24	0.1138	6.4433		0.3624		1.4101	
	TRF	24	0.3954	6.5316	NA	0.1623	NA	14.02	NA
IGF-1	Control	24	0.1426	145.0042		8.0576		1.3033	
	TRF	24	0.2409	142.8235	NA	4.8449	NA	8.2451	NA

Table S2. Circadian parameters for serum measures in urethane-administrated mice related to Fig. S3H-K.

Statistical analysis for Fig. S3H to K.									
		JTK_ pvalue	JTK_ BH.Q	JTK_ period	JTK_ adjphase	JTK_ amplitude	meta2d_ Base	meta2d_ AMP	meta2d_ rAMP
HDL-C	SNC	0.0044	0.0175	24	16	0.5478	3.4609	0.4195	0.1212
	STRF	0.3602	0.4803	24	10	0.1874	2.8279	0.1732	0.0612
	UNC	0.1834	0.3667	24	16	0.3028	3.0725	0.1326	0.0432
	UTRF	0.0012	0.0050	24	16	0.3460	2.8365	0.3064	0.1080
LDL-C	SNC	1	1	24	13	0.0147	0.3849	0.0534	0.0534
	STRF	0.5009	0.5009	24	7	0.0515	0.3519	0.0041	0.0041
	UNC	0.0020	0.0082	24	10	0.1397	0.3850	0.1703	0.1703
	UTRF	0.0490	0.0980	24	7	0.1361	0.3528	0.0225	0.0225
TCHO	SNC	0.0323	0.0647	24	19	0.2969	2.8697	0.1032	0.0360
	STRF	0.3602	0.4803	24	13	0.5567	2.7258	0.6080	0.2230
	UNC	1	1	24	1	0.1113	2.8303	0.2850	0.1007
	UTRF	0.2033	0.2033	24	13	0.2041	2.3962	0.4180	0.1744
TG	SNC	1	1	24	19	0.0579	1.0549	0.0205	0.0194
	STRF	0.2857	0.4803	24	4	0.1448	0.8562	0.2329	0.2329
	UNC	1	1	24	1	0.0676	0.9848	0.0899	0.0899
	UTRF	0.1756	0.2033	24	4	0.1835	0.8603	0.0676	0.0676

Table S3. Statistical analysis of circadian parameters related to Fig. S4.

Statistical analysis for Fig. S4									
Measures	Group	Period	Rhythmicity (pVal)	Mesor	Mesor (pVal)[a]	Amplitude	Amplitude (pVal)	Phase	Phase (pVal)
Cyclic AMP	Control	24	0.032	1338.007		460.888		14.053	
	TRF	24	0.228	1311.842	NA	209.336	NA	18.788	NA
Succinate	Control	24	0.0026	1738973.094		730974.8345		14.0173	
	TRF	24	0.0272	2083032.275	0.239	820735.4083	0.8253	16.3579	0.2545
Thiamine pyrophosphate	Control	24	0.0017	284307.2008		49642.787		17.0473	
	TRF	24	0.0262	292709.47	0.6021	41526.1582	0.7183	16.7123	0.8653
Lactate	Control	24	0	5261181.698		1115802.379		15.4921	
	TRF	24	0.0036	5428654.516	0.4348	726295.905	0.1982	14.9838	0.7135
Adenosine-monophosphate	Control	24	0	282436.4301		98095.1929		16.6332	
	TRF	24	0.0005	295556.507	0.5284	91846.8546	0.83	16.9926	0.7648
D-Fructose 1,6-bisphosphate	Control	24	0.2546	336795.7851		56388.4864		21.6105	
	TRF	24	0.0298	394261.2049	NA	124189.0438	NA	17.3892	NA
3-Phospho-D-glycerate	Control	24	0.2149	6045845.215		1049739.557		21.4809	
	TRF	24	0.2173	5914563.443	NA	1116127.823	NA	12.0016	NA
Glycerone-P	Control	24	0.1405	7660858.396		2033651.276		6.7864	
	TRF	24	0.1468	8973360.493	NA	1789614.184	NA	6.7277	NA
2-Oxoglutarate	Control	24	0.0443	1406849.513		103128.3154		7.4837	
	TRF	24	0.1324	1647924.23	NA	189178.0796	NA	2.5621	NA
Fumarate	Control	24	0.0164	4348687.774		380061.0847		6.3185	
	TRF	24	0.0231	4799906.502	0.0443	671476.6642	0.3429	3.3606	0.2437
L-Malic acid	Control	24	0.18	204230014.1		5469682.293		7.1336	
	TRF	24	0.0474	218776907.1	NA	18663462.6	NA	2.9391	NA
Oxaloacetate	Control	24	0.0663	625097.9583		31894.9635		11.3798	
	TRF	24	0.0025	654297.6463	0.0915	59248.5468	0.2666	9.3349	0.3692
Flavin mononucleotide	Control	24	0.523	63832.7677		1358.9411		6.9818	
	TRF	24	0.0178	68125.2018	NA	6441.8778	NA	8.6317	NA
Citrate	Control	24	0.0002	193026074		13797015.85		5.036	
	TRF	24	0	198196724.4	0.0933	20191237.47	0.1398	6.2048	0.2486
Adenosine 5'-triphosphate	Control	24	0.1422	601778.5771		75275.8836		18.3915	
	TRF	24	0.0498	583880.8115	NA	68718.0104	NA	14.4155	NA
NADP	Control	24	0.1072	119563.6866		28404.5007		23.2553	
	TRF	24	0.3114	107777.9253	NA	11606.448	NA	20.2293	NA
NAD	Control	24	0.2866	22321.6195		1314.4048		6.492	
	TRF	24	0.3299	22266.9417	NA	2533.8007	NA	23.4509	NA
D-Glucose 6-	Control	24	0.0001	32630978.59		7532101.921		5.1754	

phosphate	TRF	24	0.0005	38285300.41	0.0122	10655679.56	0.3113	5.6027	0.7515
Beta-D-Fructose 6-phosphate	Control	24	0.0001	22795671.85		8893670.099		4.6794	
	TRF	24	0.0004	27986647.85	0.0289	11281049.6	0.4686	6.5069	0.1502
Phosphoenolpyruvate	Control	24	0.0644	16630671.04		4934219.736		3.6179	
	TRF	24	0.0498	15717610.41	NA	5459610.764	NA	7.5339	NA
Isocitrate	Control	24	0.0064	224801.9446		100084.3131		1.6323	
	TRF	24	0.0433	393242.9114	0.0018	146544.4083	0.5297	7.9807	0.0081
cis-Aconitate	Control	24	0.0078	1837109.337		1022494.186		2.1402	
	TRF	24	0.0479	3668236.989	0.0018	1569447.978	0.4963	7.5796	0.029
ADP	Control	24	0.1346	673141.5775		448560.2731		2.2207	
	TRF	24	0.0491	756522.9557	NA	534954.6865	NA	1.0032	NA

Table S4. Statistical analysis of circadian parameters for genes expression related to Fig. 4D-I, K.

Statistical analysis for Fig. 4D-I, K									
Measures	Group	Period	Rhythmicity (pVal)	Mesor	Mesor (pVal)a	Amplitude	Amplitude (pVal)	Phase	Phase (pVal)
A549 Tumor tissue: HK1	Control	24	0.1149	2.0962		0.5027		11.2489	
	TRF	24	0.0010	1.8126	NA	0.8004	0.4262	7.6896	NA
A549 Tumor tissue: GPI	Control	24	0.0110	2.0781		0.7224		14.8747	
	TRF	24	0.0162	1.5839	0.0256	0.4132	0.3186	10.061	0.0372
A549 Tumor tissue: FBP1	Control	24	0.0000	0.6473		0.4941		5.6939	
	TRF	24	0.0000	0.5042	0.0494	0.5143	0.8363	4.1541	0.0549
A549 Tumor tissue: PFKP	Control	24	0.0167	2.3802		0.9607		13.4125	
	TRF	24	0.0209	2.6067	0.5534	1.0101	0.9280	9.1603	0.0439
A549 Tumor tissue: ALDOA	Control	24	0.0000	3.0708		2.6344		16.0659	
	TRF	24	0.0003	3.7966	0.1361	2.4544	0.7891	15.159	0.3806
A549 Tumor tissue: PGK1	Control	24	0.0017	2.3824		1.1349		15.0895	
	TRF	24	0.0162	1.7304	0.0178	0.5342	0.1205	11.8794	0.1257
A549 Tumor cell: GPI	Control	24	0.0032	1.2004		0.1845		10.4234	
	TRF	24	0.0164	0.9502	0.0002	0.1770	0.9292	6.7059	0.0452

Table S5. Statistical analysis of circadian parameters for genes levels related to Fig. 5.

**Statistical analysis for Fig. 5**									
Measures	Group	Period	Rhythmicity (pVal)	Mesor	Mesor (pVal)[a]	Amplitude	Amplitude (pVal)	Phase	Phase (pVal)
A549 Tumor cell: CRY1	Control	24	0.0000	1.079		0.4283		5.373	
	TRF	24	0.0000	0.6088	0.0000	0.3432	0.3825	3.1623	0.0296
A549 Tumor cell: CRY2	Control	24	0.1295	1.2594		0.1645		15.8802	
	TRF	24	0.0091	1.0512	NA	0.2968	NA	21.7165	NA
A549 Tumor cell: FBXL3	Control	24	0.0148	1.1462		0.3434		22.3627	
	TRF	24	0.0189	0.9350	0.0629	0.2404	0.5103	21.3518	0.6369
A549 Tumor cell: TIM	Control	24	0.0027	1.0160		0.2490		9.4774	
	TRF	24	0.0000	0.6282	0.0000	0.3410	0.3157	21.1762	0.0000
A549 Tumor cell: GPER1	Control	24	0.0021	1.1989		0.3241		16.6512	
	TRF	24	0.0020	1.2208	0.8431	0.4815	0.3196	17.5517	0.5686
Tumor tissue: CRY1	Control	24	0.0046	3.1283		1.5969		16.9627	
	TRF	24	0.0110	3.2845	0.7374	1.2183	0.5690	8.4649	0.0000
Tumor tissue: CRY2	Control	24	0.2671	1.3897		0.2052		10.415	
	TRF	24	0.0000	1.1289	NA	0.7948	NA	5.2394	NA
Tumor tissue: FBXL3	Control	24	0.0000	5.1348		4.0434		15.7851	
	TRF	24	0.0894	4.9195	NA	0.8585	NA	14.5949	NA
Tumor tissue: TIM	Control	24	0.0024	1.2889		0.4487		16.8358	
	TRF	24	0.0012	1.3770	0.5664	0.6233	0.4176	6.6902	0.0000
Tumor tissue: GPER1	Control	24	0.0001	0.8563		0.6221		5.8573	
	TRF	24	0.0001	0.7329	0.384	0.7805	0.4195	5.6279	0.8379

Table S6. Statistical analysis of circadian parameters related to Fig. S7.

**Statistical analysis for Fig. S7**									
Measures	Group	Period	Rhythmicity (pVal)	Mesor	Mesor (pVal)[a]	Amplitude	Amplitude (pVal)	Phase	Phase (pVal)
H460 Tumor cell: CRY1	Control	24	0.0828	1.1469		0.1976		3.1781	
	TRF	24	0	0.6063	NA	0.6889	NA	4.9867	NA
H460 Tumor cell: CRY2	Control	24	0.0519	1.0984		0.1604		5.7733	
	TRF	24	0.0015	0.7857	NA	0.2866	NA	2.8895	NA
H460 Tumor cell: FBXL3	Control	24	0.7824	1.2996		0.0256		23.7195	
	TRF	24	0	0.5293	NA	0.4945	NA	6.8666	NA
H460 Tumor cell: TIM	Control	24	0.0141	1.1911		0.251		6.3396	
	TRF	24	0.0011	0.9157	0.0174	0.504	0.1126	2.287	0.0374
H460 Tumor cell: GPER1	Control	24	0.0044	1.4673		0.4666		10.0398	
	TRF	24	0	1.1662	0.0169	0.5392	0.6692	6.4634	0.0094
H1975 Tumor cell: CRY1	Control	24	0.0203	1.0596		0.3713		8.0623	
	TRF	24	0.0023	1.1095	0.765	0.6756	0.2027	4.1287	0.0514
H1975 Tumor cell: TIM	Control	24	0.0104	1.0862		0.1484		17.9895	
	TRF	24	0.0254	1.1956	0.0976	0.1861	0.68	6.277	0
H1975 Tumor cell: FBXL3	Control	24	0.0029	1.0347		0.3376		9.3195	
	TRF	24	0.0237	0.8265	0.0453	0.2617	0.5946	5.0544	0.0275
H1975 Tumor cell: CRY2	Control	24	0.0828	0.6933		0.121		21.9189	
	TRF	24	0.3061	1.0081	NA	0.1423	NA	7.461	NA
H1975 Tumor cell: GPER1	Control	24	0.0427	1.0881		0.2414		17.6173	
	TRF	24	0.7968	1.0187	NA	0.0449	NA	16.4449	NA
H1299 Tumor cell: CRY1	Control	24	0.0001	1.2185		0.3585		7.036	
	TRF	24	0.0021	1.2444	0.6909	0.2397	0.2029	14.2057	0
H1299 Tumor cell: CRY2	Control	24	0	0.957		0.2237		6.0214	
	TRF	24	0.0041	0.7332	0	0.1472	0.1827	4.7547	0.3119
H1299 Tumor cell: FBXL3	Control	24	0.0029	0.9996		0.2235		22.2198	
	TRF	24	0	0.77	0.0002	0.2632	0.6137	19.2143	0.0211
H1299 Tumor cell: TIM	Control	24	0.3833	1.1469		0.046		19.7527	
	TRF	24	0.0001	0.6854	NA	0.293	NA	20.3248	NA
H1299 Tumor cell: GPER1	Control	24	0.0005	1.6611		0.6108		14.6624	
	TRF	24	0.0794	1.1335	NA	0.2452	NA	22.8491	NA
PC9 Tumor cell: CRY1	Control	24	0.0093	0.8993		0.2209		17.798	
	TRF	24	0.0033	1.0442	0.0795	0.2978	0.5009	17.4129	0.8242
PC9 Tumor cell: CRY2	Control	24	0.3472	0.8501		0.0513		2.4666	
	TRF	24	0.0172	0.6881	NA	0.1472	NA	23.7187	NA
PC9 Tumor	Control	24	0.012	0.5274		0.2019		23.5614	

cell: FBXL3	TRF	24	0.0001	0.532	0.9419	0.2856	0.3524	18.3039	0.0011
PC9 Tumor cell: TIM	Control	24	0.2028	0.7933		0.1215		10.1859	
	TRF	24	0	0.6972	NA	0.4941	NA	18.6126	NA
PC9 Tumor cell: GPER1	Control	24	0.0003	1.1769		0.3333		14.1315	
	TRF	24	0.1482	0.7396	NA	0.1391	NA	20.338	NA

Table S7. Statistical analysis of circadian parameters related to Fig. S8.

Statistical analysis for fig. S8									
Measures	Group	Period	Rhythmicity (pVal)	Mesor	Mesor (pVal)[a]	Amplitude	Amplitude (pVal)	Phase	Phase (pVal)
Lung tissue: CRY1	Control	24	0.0097	1.0688		0.3405		3.7111	
	TRF	24	0	1.1149	0.6489	0.4397	0.4855	6.4211	0.0661
Lung tissue: CRY2	Control	24	0.0087	1.8598		0.8308		17.835	
	TRF	24	0.0849	1.7014	NA	0.4716	NA	16.936	NA
lung tissue: FBXL3	Control	24	0.0911	1.7623		0.5016		16.3897	
	TRF	24	0.0379	1.5813	NA	0.4778	NA	17.2928	NA
Lung tissue: TIM	Control	24	0.0026	1.3773		0.4835		12.4161	
	TRF	24	0.4848	1.2226	NA	0.0747	NA	2.5968	NA
Lung tissue: GPER1	Control	24	0.3126	2.6829		0.8498		15.4056	
	TRF	24	0.5841	2.046	NA	0.3267	NA	3.8672	NA
Liver tissue: CRY1	Control	24	0.0037	0.7817		0.4274		4.1467	
	TRF	24	0	0.8044	0.8433	0.5311	0.5243	4.6498	0.7029
Liver tissue: CRY2	Control	24	0.0074	0.9766		0.5382		19.6877	
	TRF	24	0.0032	0.6666	0.0545	0.4408	0.6678	22.3609	0.1289
Liver tissue: FBXL3	Control	24	0.4451	1.0149		0.0745		21.3764	
	TRF	24	0.0433	0.9131	NA	0.3145	NA	18.4194	NA
Liver tissue: TIM	Control	24	0.2259	0.9089		0.247		19.5861	
	TRF	24	0.0259	0.824	NA	0.2258	NA	19.9921	NA
Liver tissue: GPER1	Control	24	0.2417	0.9397		0.2778		20.0196	
	TRF	24	0.1936	1.4147	NA	0.38	NA	17.9381	NA

Table S8. The sequences of all primers.

human-actb- FORWARD	CCTGGCACCCAGCACAAT
human-actb- REVERSE	GGGCCGGACTCGTCATAC
human-Cry2-FORWARD	CTATGAGAGACCCCGAATGAAC
human-Cry2-REVERSE	CCGCTTCACCTTTTTATACAGG
human-Fbxl3-FORWARD	GCTTAAGAGAACTAGCCCTGAA
human- Fbxl3-REVERSE	CATCCCAGCTACTCTTCTGAAT
human-Gper1-FORWARD	GAGAACGTCTTCATCAGCGT
human- Gper1-REVERSE	AAGCTGTAGATGAGGGGGTTTA
human-Cry1-FORWARD	GACGCAGCTATTAAGAAACTGG
human-Cry1-REVERSE	TTTGCTGATGAGAGTCTGGAAT
human-Tim-FORWARD	AAACAAATCCTCTCTGCTCCACTTCC
human-Tim-REVERSE	CTCCTCCTCCTCTTCTTCTTCCTCTG
mouse-actb-FORWARD	TGTCACCAACTGGGACGATA
mouse-actb- REVERSE	GGGGTGTTGAAGGTCTCAAA
mouse-Cry2-FORWARD	GAGAACTCTCACACCCTCTATG
mouse-Cry2-REVERSE	CATAGGTGTCGTCATGGTTCTC
mouse-Fbxl3-FORWARD	AAACTCTCGGACTTATCTCGAC
mouse-Fbxl3-REVERSE	GGGACTTGGAGTTTACAAACAC
mouse-Gper1-FORWARD	GAAAGCCCTGAGGATGATCTTC
mouse-Gper1-REVERSE	GTAGGTGGACACTGATGAAGAC
mouse-Cry1-FORWARD	GAGGCAAGCAGACTGAATATTG
mouse-Cry1-REVERSE	CCCATTAGAGTTAGAAGGGACC
mouse-Tim-FORWARD	AATATCACAGCCAAACGTTCAC
mouse-Tim-REVERSE	CAGCTTCTTTCTCCGTTTCTTG

TIM blots of Fig 6A

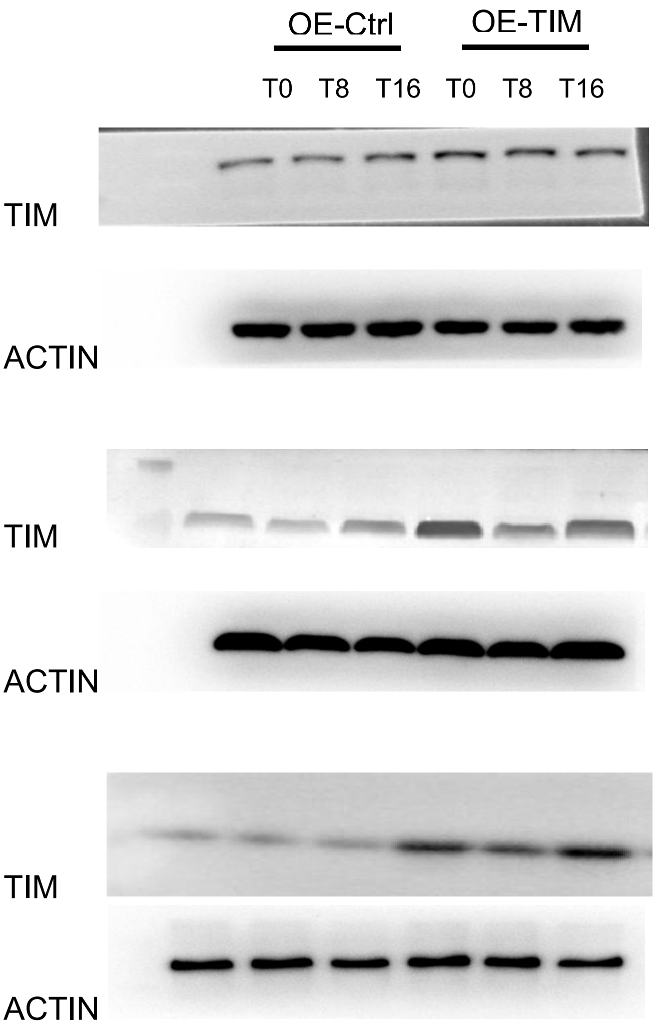

LC3B blots of Fig 7B

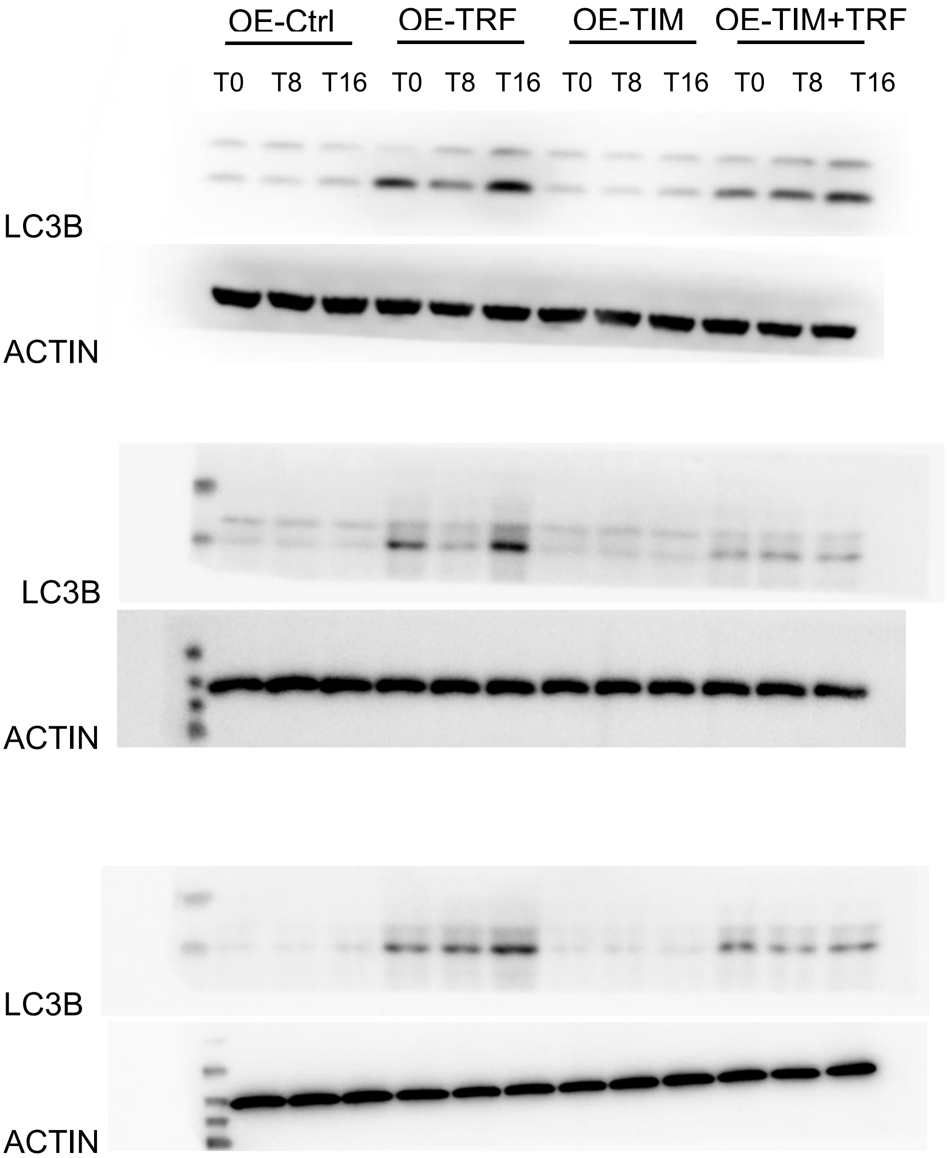

P62 blots of Fig 7B

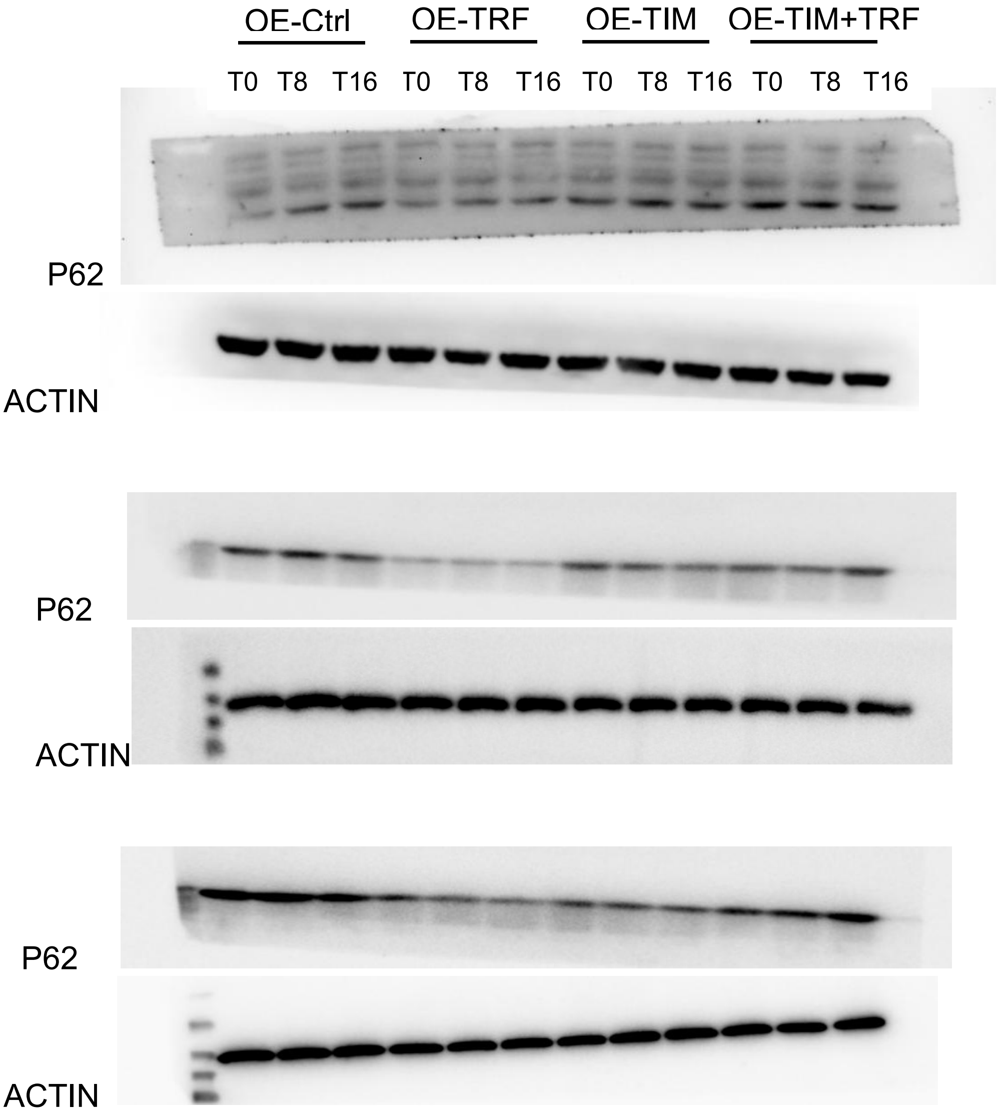

TIM blots of Fig 8A

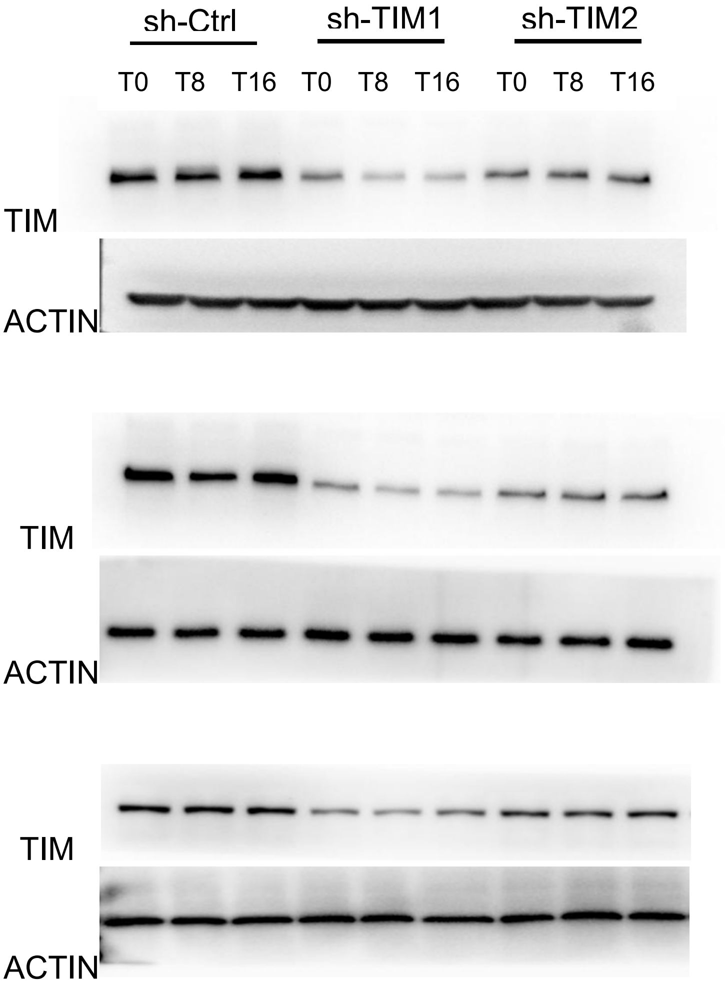

Supplement: Supplementary file 1 — Additional file 1: Fig. S1. TRF inhibits proliferation and migration of lung adenocarcinoma cells. Fig. S2. TRF effects on organ pathology in A549 xenograft lung tumorigenesis mouse models. Fig. S3. TRF effects on rhythm expression of serum measures in A549 xenograft mice. Fig. S4. TRF effects on temporal expressions of metabolites involved in energy metabolism. Fig. S5. GPI is not involved in TRF-mediated anti-tumor effect in vitro. Fig. S6. Clock genes expressions are altered in lung adenocarcinoma and correlate with survival phenotype. Fig. S7. TRF regulates the rhythm expression of circadian genes in multiple tumor cell lines. Fig. S8. The effects of TRF on clock genes in the lung and liver tissues. Table S1. Circadian parameters for serum measures in xenograft lung tumorigenesis mice related to Fig. S3A-G. Table S2. Circadian parameters for serum measures in urethane-administrated mice related to Fig. S3H-K. Table S3. Statistical analysis of circadian parameters related to Fig. S4. Table S4. Statistical analysis of circadian parameters for genes expression related to Fig. 4D-I, K. Table S5. Statistical analysis of circadian parameters for genes levels related to Fig. 5. Table S6. Statistical analysis of circadian parameters related to Fig. S7. Table S7. Statistical analysis of circadian parameters related to Fig. S8. Table S8. The sequences of all primers. [file 12916_2023_3131_MOESM1_ESM.pdf]
